# Topographic precision in sensory and motor corticostriatal projections varies across cell type and cortical area

**DOI:** 10.1038/s41467-018-05780-7

**Published:** 2018-09-03

**Authors:** Bryan M. Hooks, Andrew E. Papale, Ronald F. Paletzki, Muhammad W. Feroze, Brian S. Eastwood, Jonathan J. Couey, Johan Winnubst, Jayaram Chandrashekar, Charles R. Gerfen

**Affiliations:** 1 Department of Neurobiology, University of Pittsburgh School of Medicine, Pittsburgh, PA USA; 2 Laboratory of Systems Neuroscience, NIMH, Bethesda, MD USA; 3 MBF Bioscience, Williston, VT USA; 4 Janelia Research Campus, Ashburn, VA USA

## Abstract

The striatum shows general topographic organization and regional differences in behavioral functions. How corticostriatal topography differs across cortical areas and cell types to support these distinct functions is unclear. This study contrasted corticostriatal projections from two layer 5 cell types, intratelencephalic (IT-type) and pyramidal tract (PT-type) neurons, using viral vectors expressing fluorescent reporters in Cre-driver mice. Corticostriatal projections from sensory and motor cortex are somatotopic, with a decreasing topographic specificity as injection sites move from sensory to motor and frontal areas. Topographic organization differs between IT-type and PT-type neurons, including injections in the same site, with IT-type neurons having higher topographic stereotypy than PT-type neurons. Furthermore, IT-type projections from interconnected cortical areas have stronger correlations in corticostriatal targeting than PT-type projections do. As predicted by a longstanding model, corticostriatal projections of interconnected cortical areas form parallel circuits in the basal ganglia.

## Abstract (web summary)

How corticostriatal connections of different pyramidal cell types are organized, particularly in convergent circuits, has not been evaluated in detail. Here, cell type-specific Cre-driver mice reveal that pyramidal tract-type corticostriatal projections, though broadly similar to intratelencephalic-type projections from the same cortical region, are generally more restricted and variable in their topographic termination patterns.

## Introduction

Primary motor (M1) and primary somatosensory (S1) areas of cerebral cortex are somatotopically organized, with distinct body regions represented in adjacent areas. Though sensory and motor cortices specialize in distinct functions, corticocortical projections reciprocally connect them. Similarly, corticostriatal inputs are topographically organized. Overlaid on this pattern, however, output from any given cortical area projects broadly and overlaps with output from other areas, including topographically related ones^1,2^. A longstanding model of corticostriatal organization is that striatal regions integrate input from multiple cortical areas that are functionally interconnected^3,4^. This suggests that the striatum is organized into distinct regions^2^ associated with different behavioral functions^5,6^. While there is topographic organization, different functions of dorsolateral, dorsomedial, and ventral divisions are not rigidly spatially segregated^7,8^. To better understand how information from the cortex is integrated within the striatum, this study first asks whether projections from different cortical areas project to stereotyped sectors of striatum across animals by quantifying overlap and segregation between sensory, motor, and frontal projections. Stereotypy refers to the degree to which injections from a given brain location project to the same target location across different animals. As a subsequent step, these data tests whether corticocortical connectivity predicts convergence or interdigitation within the striatum.

Addressing these questions is not straightforward with conventional anatomical techniques, since the corticostriatal projection originates from two distinct excitatory neuron categories in layer 5 (L5): pyramidal tract-type (PT-type) neurons and intratelencephalic (IT-type) neurons^9–11^. PT-type neurons send projections to the thalamus, subthalamic nucleus, superior colliculus, and brainstem with collaterals in ipsilateral striatum^12^, but do not project to contralateral cortex nor contralateral striatum. In contrast, IT-type cells project exclusively to ipsi- and contralateral striatum and cortex, and not to other subcortical targets^11^. In motor areas, local circuits are hierarchically organized such that IT-type cells connect to each other and project to PT-type neurons, but PT-type neurons do not connect to IT-type cells^13^. Thus, information at different stages of processing is transmitted out of cortex, conveying distinct messages^14^.

The differences between the corticostriatal projections of these two major cell types are analyzed using stereotaxic injection of Cre-dependent reporters into sensory, motor, and frontal cortical areas of Cre-driver mice selective for IT-type and PT-type neurons. Sectioned brains are then imaged and aligned to a reference brain, the Mouse Common Coordinate Framework version 3 (CCF v3)^15–17^ to quantify axonal fluorescence in a standard coordinate system. Targeting of axonal projections in striatum and other targets of motor and sensory output is quantified to assess the topographic organization of projections. These data reveal that the topographic organization of projections differs between IT-type and PT-type neurons and between sensory and motor areas. Thus, the information cortex provides for striatal processing differs across these two cortical output channels.

## Results

### A library of IT-type and PT-type corticostriatal projections

**Fig. 1:**
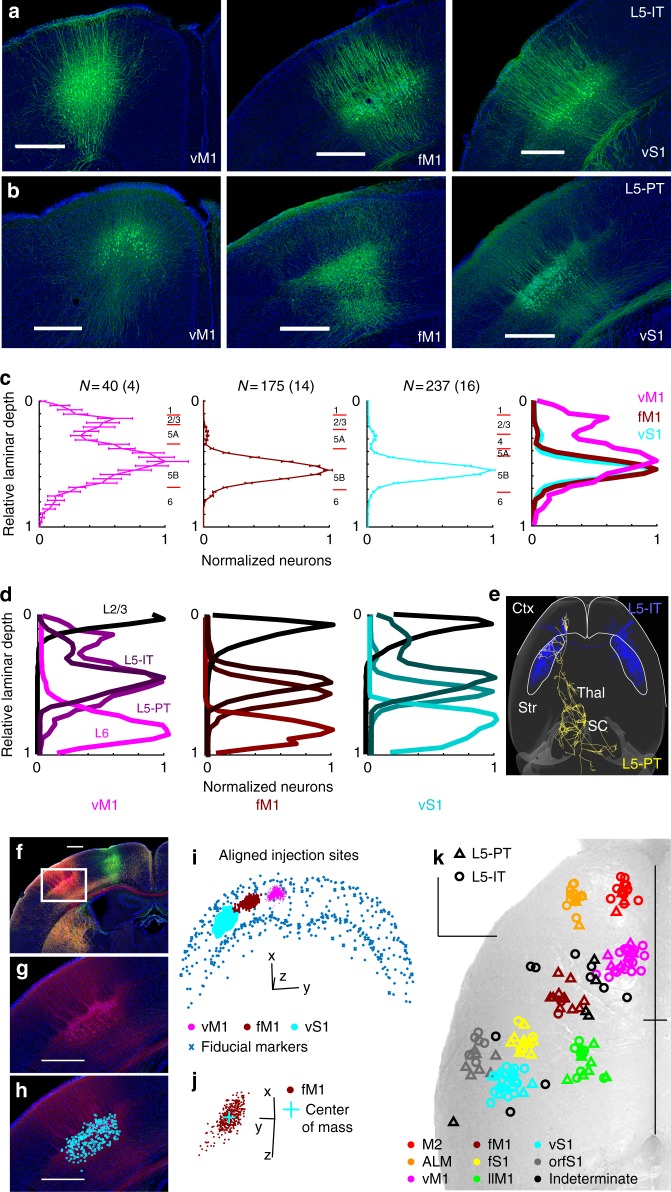
Cre-driver mice label specific pyramidal neuron types. **a**, **b** Coronal images of injection sites in Tlx3_PL56 (L5-IT) and Sim1_KJ18 (L5-PT) show somata locations. Scale, 0.5 mm. **c** Somata locations of Sim1_KJ18 mice injected in vM1, fM1, and S1. Comparison at right. N indicated shows # of sections (# of mice). Purple, vM1; burgundy, fM1; teal, vS1. Pia is relative laminar depth of 0; white matter is 1. Error bars represent SEM. Red tick marks show estimated cortical layers. **d** Mean somata distribution for lines labeling L2/3 (Sepw1_NP39), L5-IT (Tlx3_PL56), L5-PT (Sim1_KJ18), and L6 (Ntsr1_GN220). **e** Targets of IT-type (Tlx3_PL56) and PT-type (Sim1_KJ18) neurons, illustrated with single-axon reconstructions. IT-type neurons (blue) project to ipsi- and contralateral cortex (Ctx) and striatum (Str). PT-type neurons (gold) target ipsilateral cortex, striatum, and subcortical targets in thalamus (Thal), superior colliculus (SC) and brainstem. **f**–**h** Image of Sim1_KJ18 injection site. White box in **f** indicates magnified area (**g**, **h**). Scale 0.5 mm. **h** Annotation of somata in Neurolucida. Blue circles indicate AAV-infected cell bodies expressing smFPs. **i**, **j** Coordinates of somata and fiducial markers placed along pia and white matter of cortex were aligned to the CCF. Somata for three injections (teal, burgundy, and purple) and fiducial markers (gray) were then plotted in 3-d (1 mm scale). Rotated projection (**j**) shows dorsal view of injection site center of mass (teal) and anterior/posterior spread of infection. (k) Injection site center of mass of Tlx3_PL56 (*N* = 92, circles) and Sim1_KJ18 (*N* = 62, triangles) plotted and spatially clustered. Eight clusters shown in red (M2), orange (ALM), purple (vM1), burgundy (fM1), green (llM1), yellow (fS1), teal (vS1), and gray (orfS1). Indeterminate injection sites are black. Sites are superimposed on an image of the dorsal surface of mouse cortex. Black cross marks midline and bregma. Scale, 1 mm

To analyze the corticostriatal projections of specific pyramidal cell types, mouse lines selectively expressing Cre in IT-type (Tlx3_PL56) and PT-type (Sim1_KJ18) neurons^18^ were injected with AAV expressing Cre-dependent tracers. Each mouse received injections of three different AAV vectors (GFP, td-tomato, and smFPs; Table 1^19^) into different locations of sensory, motor and frontal cortex (Fig. 1 and Supplementary Fig. 1). A whole-brain reconstruction from tiled images^20^ (Supplementary Fig. 1b-e) was registered to a common reference frame using BrainMaker software (MBF Bioscience) with alignment precision of ~50–70 µm (Supplementary Fig. 1l-y). Original images were posted at: http://gerfenc.biolucida.net/link?l=Jl1tV7 (Biolucida viewer-based, free download) and http://gerfenc.biolucida.net/images/?page=images&selectionType=collection&selectionId=32  (web-based). Placing all voxels from all brains in the same reference space enabled quantitative analysis of regions of interest across different animals (Supplementary Fig. 1h-i).

**Table 1:** Constructs for tracing

Construct name	Addgene number	Addgene name	Penn Vector Core number	Penn Vector Core name
AAV2/1-CAG-flex-EGFP	51502	pCAG-FLEX-EGFP-WPRE	AV-1-ALL854	AAV1.CAG.Flex.eGFP.WPRE.bGH
AAV2/1-CAG-flex-tdTomato	51503	pCAG-FLEX-tdTomato-WPRE	AV-1-ALL864	AAV1.CAG.Flex.tdTomato.WPRE.bGH
AAV2/1-CAG-flex-GFPsmFP-FLAG	59756	pCAG-smFP-FLAG	—	—
AAV2/1-CAG-flex-GFPsmFP-Myc	59757	pCAG-smFP-Myc	AV-1-PV3511	AAV1.CAG.GFPsm-myc.WPRE.SV40
AAV2/1-CAG-flex-GFPsmFP-V5	59758	pCAG-smFP-V5	—	—
AAV2/1-CAG-flex-GFPsmFP-HA	59759	pCAG-smFP-HA	—	—
AAV2/1-CAG-flex-mRuby2smFP-FLAG	59760	pCAG-mRuby2-smFP-FLAG	AV-1-PV3509	AAV1.CAG.Ruby2sm-FLAG.WPRE.SV40
AAV2/1-CAG-flex-mRuby2smFP-OLLAS	59761	pCAG-mRuby2-smFP-OLLAS	—	—

As expected for IT-type neurons, injections in Tlx3_PL56 mice labeled axonal projections that bilaterally targeted cortex and striatum, but not other subcortical structures^10,11^ (Fig. 1e). By contrast, axonal projections in the Sim1_KJ18 line were restricted to the hemisphere ipsilateral to the injection within the cortex and striatum. Labeled neurons also projected to the thalamus, subthalamic nucleus, superior colliculus, pontine and medullary nuclei, typical of PT-type corticofugal neurons^12^. IT-type neurons are generally located in more superficial layer 5 than PT-type neurons, with considerable overlap. Injections in Sim1_KJ18 and Tlx3_PL56 infected a small number of L2/3 neurons. Somata of labeled pyramidal neurons at injection sites were marked in Neurolucida and their relative laminar depth plotted (Fig. 1a-d). Tlx3_PL56 and Sim1_KJ18 labeled neurons at injection sites were consistent with prior descriptions of the laminar locations of IT and PT neurons^21,22^. As subtypes of PT-type neurons exist, targeting either thalamus or medulla, for example, PT-type neurons of Sim1_KJ18 represent a mixture of these subpopulations since projections can be seen in a range of subcortical targets^23^. Similarly, IT-type neurons of Tlx3_PL56 project to multiple expected ipsi- and contralateral cortical and subcortical targets, which may be individually targeted by more finely defined subsets of IT-type neurons.

The coordinates of labeled somata for each injection in the original images were marked and transformed into the CCF reference frame (Fig. 1f-k), with the average used to determine a center of mass for the injection site (Fig. 1j). The injection site center of mass was used to cluster injection sites for Sim1_KJ18 and Tlx3_PL56 into eight clusters across sensory, motor, and frontal cortex (Fig. 1k). These corresponded to vibrissal, forelimb, and orofacial somatosensory cortices (vS1, fS1, and orfS1); vibrissal, forelimb, and lower limb motor cortices (vM1, fM1, and llM1); and frontal areas (anterior lateral motor cortex (ALM) and secondary motor cortex (M2)). Indeterminate injection sites (black) were not clustered. The names assigned to these sites correspond to the body representation determined by microstimulation mapping for motor areas^24,25^ and somatotopic mapping of sensory areas^26–28^. Thus, vM1 is the low-threshold region where microstimulation evokes whisker movement and also the region where vS1 axons project. This area is also called MOs in the Allen Reference Atlas^15,16^. M2 is the frontal region reciprocally connected to vM1^29^, with ALM lateral to it.

**Fig. 2:**
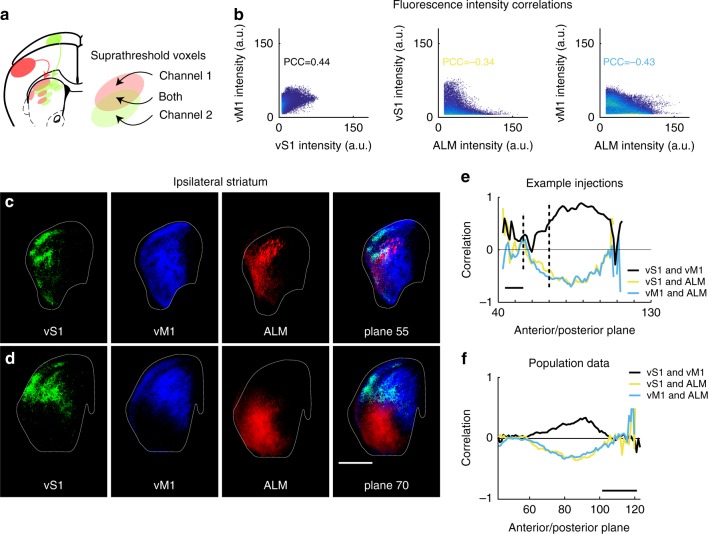
Computation of correlation for projections to ipsilateral striatum. **a** Correlation for two projections (red and green channels) in a given structure (striatum, illustrated) is computed based on voxels where both channels are suprathreshold. **b** For three example Tlx3_PL56 (IT-type) mouse injections in vM1, vS1, and ALM, scatterplot of all voxel intensities (8-bit imaging; arbitrary units) in ipsilateral striatum for two injections. Blank space between axis and points indicates threshold. Individual points in dark blue; multiple points increase yellow intensity. **c**, **d** Example coronal images from aligned brains in corresponding planes showing arborization of IT-type axons in ipsilateral striatum. vS1 shown in green, vM1 in blue, and ALM in red. Scale, 1 mm. **e** Correlation coefficient as a function of anterior/posterior plane in ipsilateral striatum for pairwise comparisons between three example injections. Correlation is noisy at anterior and posterior poles of striatum due to small voxel numbers in those planes. Scale, 1 mm. **f** Population mean correlation coefficient as a function of anterior/posterior plane in ipsilateral striatum for pairwise comparisons (the mean correlation for each vS1 compared to each vM1 in black, for example). vS1 and vM1 comparison, *N* = 340 injection pairs; vS1 and ALM comparison, *N* = 204; vM1 and ALM comparison, *N* = 240. Scale, 1 mm

A methodology was developed to quantitatively compare projections from different injections sites. Images were thresholded to eliminate 99% of background (Supplementary Fig. 1z). Three example injection sites (from Tlx3_PL56 mice in vM1, vS1, and ALM) illustrate the methodology for comparison (Fig. 2). Suprathreshold voxel intensity for ipsilateral striatum was compared on a voxel-by-voxel basis using voxels that were suprathreshold for *both* channels (Fig. 2a). The Pearson correlation coefficient (PCC) based on voxel intensity (AU) was used to assess the relationship within the striatum for each pair of injections (Fig. 2b). To localize where within the striatum correlations occurred, correlation was computed and plotted for each plane along the anterior/posterior axis (Fig. 2c-e). Correlation values varied dependent on both the particular injection site locations and the rostro-caudal level of the striatum. In the example shown, correlation was near zero in anterior striatum, but became well correlated for vS1 and vM1 in mid- and posterior ipsilateral striatum (black line). In contrast, correlation is negative for both vS1 and vM1 when compared to the ALM injection (yellow and blue lines, Fig. 2e, f). Correlations were noisier when measured based on small numbers of voxels (anterior and posterior poles of striatum, Fig. 2e, f). The general pattern was similar for individual pairs of injection cases (Fig. 2e; e.g., vS1 & vM1) compared to the population average across all pairs of injection cases at similar loci (Fig. 2f), but the magnitude of correlation varied considerably depending on individual M1 and S1 injection cases considered. In addition to corticostriatal terminal arborizations, there are some bright, dense fascicles specific to PT-type axons which pass through striatum without terminating. These could not easily be separated from the signal of terminal arborizations by thresholding, and thus contribute to the noise of this measurement.

The anatomical overlap of terminal arborizations, however, corresponds to shared targeting of functional synaptic output to specific single neurons. This was tested using a dual channel circuit mapping approach with conventional ChR2 and red-shifted ReaChR^30^ expressed in vM1 and vS1 respectively. Whole cell recordings from striatal projection neurons (SPNs) in the overlapping region of vM1 and vS1 projections revealed synaptic convergence in all neurons recorded (Supplementary Fig. 2). This confirmed that convergent axonal projections, such as those from topographically aligned regions of sensory and motor cortex, also shared functional synaptic targets.

### Topography of sensory and motor corticostriatal projections

**Fig. 3:**
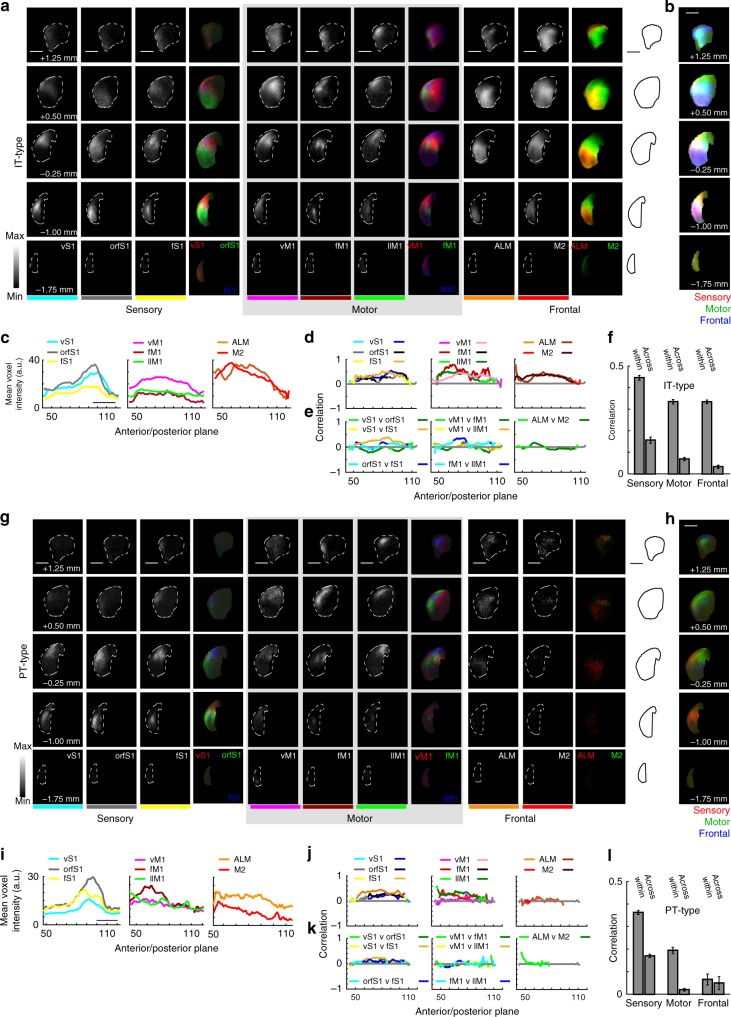
Topography of corticostriatal projections from IT-type and PT-type neurons. **a** Images of average corticostriatal IT-type projections. Rows represent images at five coronal planes from anterior (+1.25 mm to bregma) to posterior (−1.75 mm). Scale, 1 mm (all panels). Dashed line outlines ipsilateral striatum. Voxels are 50 × 50 × 50 µm. Columns represent eight injection site clusters, organized into sensory, motor, and frontal modalities. Images show average normalized projections for a given cluster. These are color coded and presented at right to show within-modality topography. vS1 projections (red) are more dorsal than orfS1 (green). **b** Average normalized sensory (red), motor (green), and frontal (blue) projections illustrate topography across modalities. **c** Mean voxel intensity along the anterior/posterior axis. Planes are 50 µm. Injection site clusters are color coded. **d** Within-cluster comparisons show high correlation for nearby injections in the same cluster. The left (sensory) plot shows mean correlation for a given vS1 injection compared to other vS1 injections. Two colors are used (left, teal; right, blue) with the right-hand color indicating locations along the anterior/posterior axis where correlation is significantly different from shuffled data (*p* < 0.001, rank sum test). Two colors are used for each injection site cluster (legend at top; the right color marks significant differences). Similar comparison performed for all eight clusters. Comparisons made in planes with > 100 suprathreshold voxels. **e** Across cluster comparisons compare injections within the same modality. For sensory clusters, vS1 injections are compared to orfS1(green) and fS1 (yellow), and orfS1 injections are compared to fS1 (blue). Across cluster comparisons are made for motor and frontal cases. **f** Mean correlations within (vS1-vS1) and across (vS1-orfS1, etc.) ipsilateral corticostriatal projections from IT-type pyramidal neurons. Correlations within a given cluster are greater than correlations across nearby clusters. **g**–**l** Analysis for PT-type projections, presented as for IT-type projections

To study topography of ipsilateral corticostriatal projections, the analysis of corticostriatal correlations was extended to all eight injection clusters, which included sensory areas (vS1, fS1, and orfS1), motor areas (vM1, fM1, and llM1), and frontal areas (ALM and M2). Sensory, motor, and frontal areas were taken to represent three modalities for cortical function, with the clusters within each modality representing different somatotopic regions (whisker, forelimb, and hindlimb for example) within that modality. First, IT-type projections were quantified. Projections from different regions within the same cortical modality displayed a topographic organization along the rostral/caudal axis (Fig. 3a; adjacent locations of red, green and blue). This demonstrated the maintenance of the topographic organization within modalities in their projections to the striatum. Comparison of the projections between sensory, motor and frontal areas showed considerable overlap (Fig. 3b; mixing of red, green, and blue). Quantitative analysis reveals varying levels of corticostriatal input along the rostro-caudal axis (Fig. 3c). Somatosensory injections were biased towards more posterior sites, with maximum intensity and suprathreshold voxel numbers peaking more caudally than motor or frontal injections.

To assess corticostriatal topography, quantitative comparisons were made between injections in the same injection cluster (Fig. 3d) or across injection sites within the same modality (Fig. 3e) using the methods described (Fig. 2). Comparison of correlation coefficients between injections within the same cluster (vS1 to other vS1 injections, Fig. 3d) showed these were always positively correlated. However, there was remarkably little correlation between injection sites across clusters within the same modality (Fig. 3e-f; Supplementary Fig. 3). ALM compared to the other frontal injection site, M2, showed near-zero correlation, as did vM1-llM1, vS1-orfS1, and orfS1-fS1 comparisons. Where there was positive correlation observed in across-cluster comparisons, this was weaker than within-cluster comparisons. This suggested stereotypy in axonal projection patterns across mice. Contralateral corticostriatal projections (Supplementary Fig. 4) had grossly similar results with weaker overall correlations. Frontal areas, however, had particularly strong contralateral projections and similarly strong within-cluster correlation.

This analysis was repeated for PT-type projections grouped into the same eight clusters by injection site location (Fig. 3g-l). There were general similarities, with frontal and motor projections targeting more anterior sites and sensory projections targeting more posterior ones. In contrast to IT-type projections, PT-type projections from frontal areas had fewer suprathreshold voxels and showed reduced mean voxel intensity compared to IT-type tracing from the same region (Fig. 3i). This reduction in intensity was consistent with smaller projections and less overlap between different injection sites. In the PT-type case, there is more sensory vs. motor separation along the anterior/posterior axis (the posterior planes have more red color, Fig. 3h). In comparison, for IT-type projections, there is relatively more overlap in posterior planes (purple posterior planes in Fig. 3b). Comparisons for nearby injection cases in the same cluster (vS1 to vS1) had higher positive correlations than comparisons to injection cases in nearby clusters, such as vS1-orfS1 or vS1-fS1 (Fig. 3j-l; Supplementary Fig. 3). The correlations for all within and across group comparisons were summarized in Fig. 3l. Correlation scores were always higher for within than across group comparisons. Furthermore, PT-type projections have lower correlations than IT-type ones (Fig. 3f, l).

### Topographic differences of IT-type and PT-type projections

**Fig. 4:**
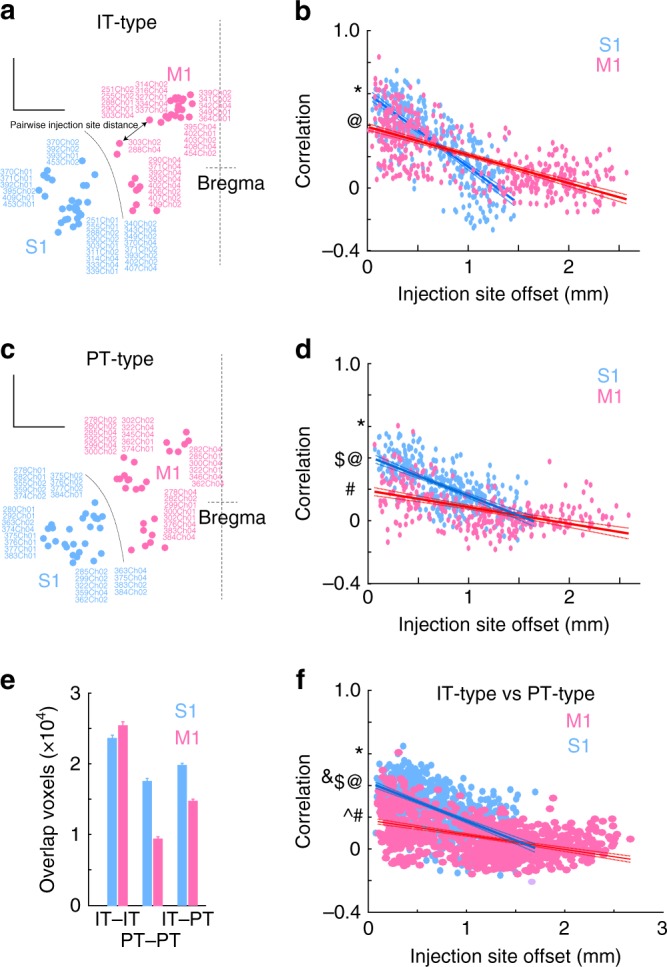
Corticostriatal topographic precision across cortical areas and cell types. **a**, **b** Pairwise correlation between ipsilateral corticostriatal projections from IT-type (**a**, **b**) and PT-type (**c**, **d**) neurons was plotted against injection site offset in mm. Scale, 1 mm. **a**, **c** Dorsal view of injection sites in CCF coordinates. Midline and bregma indicated at right. Scale bars, 1 mm. All primary sensory (S1) injections are shown in blue and primary motor (M1) injections are shown in pink. Circles indicate injection site with injection number labeled. Double headed arrow indicates injection site offset distance for one pair of injections. **b**, **d**, **f** Correlation versus injection site offset for S1 and M1 injections. *N* = 351, IT-type vs. IT-type in S1; *N* = 435, IT-type vs. IT-type in M1; *N* = 312, PT-type vs. PT-type in S1; *N* = 300, PT-type vs. PT-type in M1; *N* = 702, IT-type vs. PT-type in S1; N = 750, IT-type vs. PT-type in M1). Solid line represents linear fit, with confidence interval plotted as dashed lines. Typographic marks indicate y-intercept across panels for comparison. **e** Mean number of overlapping voxels used to calculate correlations for IT–IT (**b**), PT–PT (**d**) and IT-PT (**f**) comparisons. **f** Correlation versus injection site offset for comparisons across IT-type and PT-type injections in S1 and M1. Here, each S1 IT-type injection is compared to each S1 PT-type injection but not to other IT-type injections

Because these injections densely sampled sensory and motor areas, topographic specificity could be examined by comparing injections at a range of distances in the same or different cell types. Injection sites from different mice in the same location of the CCF are expected to share high correlation in their projections if connections in the rodent brain are stereotypical. Barrel cortex, for example, is sufficiently stereotyped that individual barrels are apparent in the Allen averaged registration template^17^. In contrast, microstimulation maps for movement show some inter-animal variability^24,25^. To examine the relationship between the distance between injection sites and their projections, the distance between injection site centers of mass was calculated for IT-type or PT-type injections in sensory and motor cortex. The correlation score in ipsilateral striatum was plotted against injection site offset (Fig. 4). For both sensory (blue) and motor injections (pink; Fig. 4b, d, f), the correlation score was fit with a linear regression (95% confidence interval shown). For IT-type projections, the peak correlation was higher for sensory cortical injections (~0.6) than for motor cortex (~0.4). The relationship dropped off more steeply in sensory areas (ANOCOVA, Group*X Value, *p* < 0.0001). Collectively, these results suggest that sensory cortical areas show stronger topographic precision than motor ones^24,25,28,31–33^. A similar relationship was apparent for PT-type projections, with higher correlations in nearby sensory injections than in motor areas (ANOCOVA, Group*X Value, *p* < 0.0001; *N* = 312, KJ18-KJ18 S1; *N* = 300, KJ18-KJ18 M1). Peak correlation was stronger for IT-type than PT-type projections for both sensory and motor populations.

**Fig. 5:**
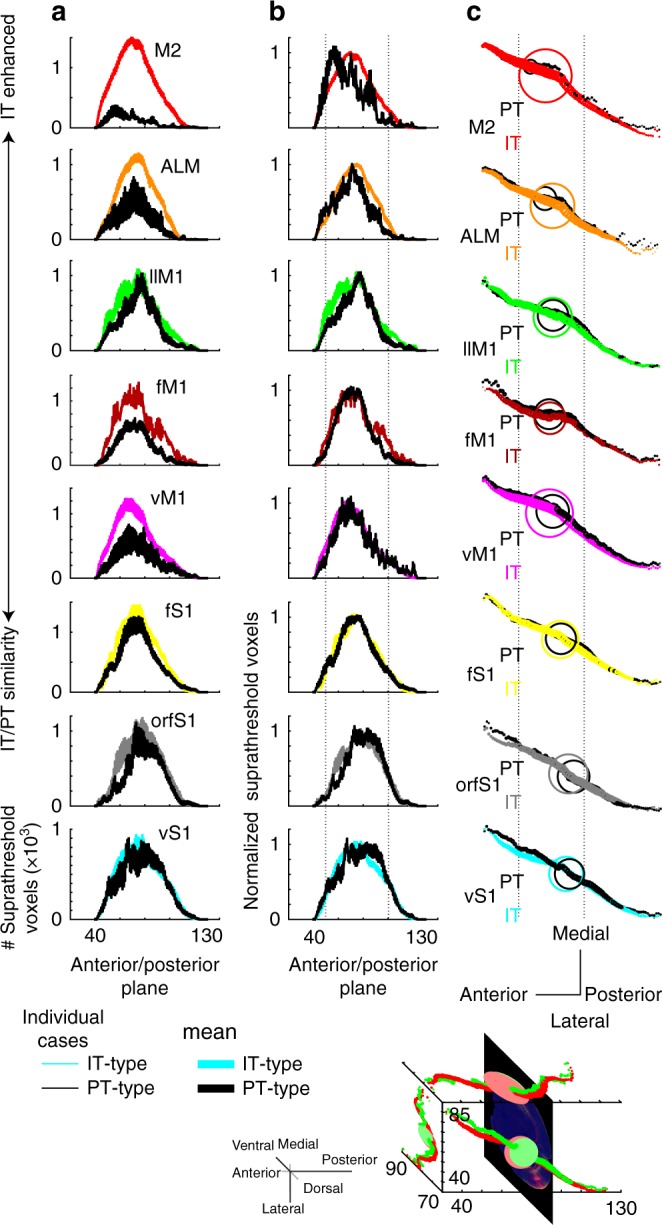
Anterior/posterior localization of IT-type and PT-type corticostriatal projections. **a** Anterior/posterior location of suprathreshold voxels in ipsilateral striatum was quantified for IT-type and PT-type injection cases. Mean ± SEM plotted. IT-type projections are highlighted in color (for example, vS1 is teal). Corresponding PT-type projections from the same cluster are plotted in black on the same axes. Number of suprathreshold voxels is similar for vS1, orfS1, and fS1 injections. Suprathreshold voxels for IT-type projections from frontal areas exceed those of PT-type projections. **b** Peak normalized distribution (Mean ± SEM) of IT-type and PT-type projections are shown. These peak at similar points on the anterior/posterior axis. **c** To assess differences in targeting of IT-type and PT-type projections within the same injection site cluster, the center of mass of corticostriatal fluorescence of the voxels for the mean normalized injection pattern was calculated for each injection site cluster. Overall center of mass is shown as a large circle (red (vS1 IT-type) and green (vS1 PT-type) circles, example at bottom). Corticostriatal fluorescence center of mass of each plane is plotted as a circle, and projections along the *x*/*y*/*z*-axes are shown. Size of the circle is proportional to the summed normalized voxel intensity for a given plane. Anterior/posterior projections for each injection cluster are shown above. Color code corresponds to the injection site cluster (teal for vS1), with IT-type projections shown in color and corresponding PT-type projections shown in black. Dotted line is shown for anterior/posterior alignment across injection clusters. Corticostriatal fluorescence center of mass of vS1, orfS1, and fS1 (teal, gray, and gold, respectively) are posterior within the striatum, while frontal areas ALM and M2 (orange and red) are anterior. Overall center of mass of corticostriatal fluorescence overlaps for IT- and PT-type cases, resulting in overlap of these markers. Scale, 1 mm (bottom right)

The correlation of IT-type with PT-type injections near the same site was also studied. If these projections targeted different striatal regions, then both a reduction in the correlation as well as a reduction in the number of overlapping voxels were expected. However, the correlation versus distance relationship was similar to that of the within PT-type injection comparisons (Fig. 4f) while the number of overlapping voxels was intermediate to IT–IT and PT–PT comparisons (Fig. 4e). Corticostriatal projections of both types tended to target the same striatal regions for a given cortical injection site, as the center of mass of corticostriatal fluorescence was generally found in the same portions of striatum (Fig. 5). Differences in these correlations could thus not be attributed to IT-type and PT-type neurons from the same cortical area targeting largely distinct striatal regions.

The departure from perfect correlation between projections from nearly overlapping injection sites could result from differences in the injection size (including number of infected cells and scatter at the injection site), inter-animal variability, or noise in image acquisition. Thus, whether different degrees of injection site scatter resulted in less correlation was tested. Injection site scatter was measured as the standard deviation for each infected soma from the injection site center of mass in a given injection. This was used to divide injections into two categories: those with scatter higher or lower than the mean. Correlation of ipsilateral striatal projections for low and high scatter groups was compared (Supplementary Fig. 5). Two populations were nearly indistinguishable, suggesting that injection size was not a major contributor to differences in correlations.

**Fig. 6:**
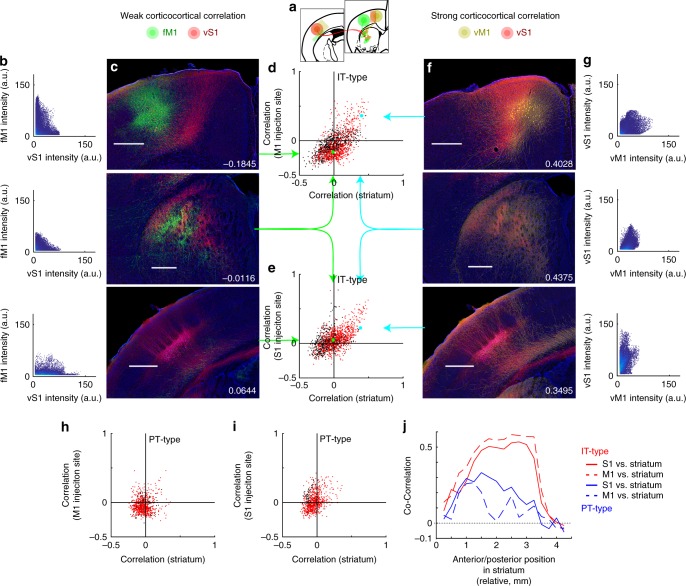
Corticostriatal projections map the organization of corticocortical connectivity in IT-type but not PT-type projections. **a** Sensory and motor cortex injections label reciprocal intracortical projections between topographically related areas. **b**, **c**, **f**, **g** IT-type injection examples shown contrast a pair of strongly connected cortical areas (red vS1 and yellow vM1 injections) with a non- topographically aligned area (green fM1 injection). Scale 0.5 mm (all panels). vS1 axons (red) overlap poorly with fM1 neurons (green). Cortical correlations are measured using fluorescence from voxels in either M1 (top) or S1 (bottom). For one pair (**b**, **c**), these are poorly correlated in both injection sites (−0.1845 and 0.0644; **b**, **c** top and bottom) as well as the striatum (−0.0116; **b**, **c** middle). In contrast, vS1 axons (red) overlap well with vM1 neurons (yellow) and are strongly correlated in both injection sites (0.4028 and 0.3495; **f**, **g** top and bottom) as well as the striatum (0.4375; **f**, **g** middle). **d**, **e** Scatterplot of co-correlations of corticocortical connectivity (using injection site overlap) and corticostriatal connectivity for IT-type projections. Each individual point represents the corticostriatal correlations (*x*-axis) and injection site correlation (*y*-axis) for a single pair of injection cases with corticocortical correlation computed at either M1 (**d**) or S1 injection sites (**e**). Red points on the scatterplot compare sensory and motor injections. Black points add comparisons to frontal areas (M2 and ALM). Green and teal arrows and points indicate specific points corresponding to the example injections shown. **h**, **i** Scatterplot of co-correlations of corticocortical connectivity and corticostriatal connectivity for PT-type projections. **j** Co-correlations of corticocortical connectivity and corticostriatal connectivity are re-assessed, with corticostriatal correlations (*y*-axis) calculated using subsets of striatal voxels along the anterior/posterior axis in 250 µm segments (*x*-axis, in mm). Co-correlation is plotted for IT-type (red) and PT-type (blue) injections

One model of corticostriatal organization suggests that striatal regions integrate input from multiple interconnected cortical areas^4^. This predicts that reciprocally connected regions of sensory and motor cortex would have elevated correlation in their striatal projections. Thus, a measure of corticocortical correlation was used to assess how well two given injection cases were reciprocally connected, and the correlation of their corticostriatal projections was used as a measure of integration in striatum. Pairwise comparisons between motor and sensory injections were thus examined for co-correlation. To assess the degree of corticocortical correlation, overlap of S1-originating axons with labeled neurons at M1 injection sites was determined (and vice-versa for M1-originating axons). The M1 and S1 injection sites were defined in the CCF using coordinates that encompassed all labeled somata at the motor or sensory injection site, and included all voxels from pia to white matter. The correlation between a pair of M1 and S1 injections was then determined in this cortical volume, using the methods described in Fig. 2. Scatterplots compare the corticocortical correlation to the corticostriatal correlation for the same pair of injection cases (green and teal arrows, Fig. 6d, e). Each point represents the comparison of a single pair of injection cases. Red points specifically highlight comparisons between sensory and motor injections. Black points label pairwise comparisons between frontal areas and either motor (Fig. 6d) or sensory cortex (Fig. 6e). For IT-type projections, there was a positive relationship for striatal comparisons to M1 and S1 injection sites (Fig. 6d, e; *R*^2^ = 0.3640 for striatal correlation vs M1 injection site correlation; *R*^2^ = 0.3055 for S1 injection site correlation). In contrast, PT-type projections did not show this strong relationship (Fig. 6h, i and Supplementary Fig. 6; *R*^2^ = 0.0038 for striatal correlation vs M1 injection site correlation; *R*^2^ = 0.1219 for S1 injection site correlation). Because IT-type corticostriatal projections generally project to a greater area in striatum (Figs 3, 4), it is possible the increased co-correlation resulted from IT-type projection overlap in a focal region not innervated by PT-type neurons. Thus, the relationship between anterior/posterior subsets of the striatum with corticocortical connectivity (measured as before) was assessed by examining the co-correlation of cortical and striatal connectivity along 250 µm striatal segments. The co-correlation is determined by the correlation of two correlation coefficients (the graphs of Figs 6d, e and 5h, I, computed for anterior/posterior subsets of striatal voxels). This revealed a long plateau of high co-correlation across the rostro-caudal extent of striatum (Fig. 6j) for IT-type but not PT-type projections. The enhanced co-correlation for IT-type projects did not result from a single focal region, but was spread across the extent of the corticostriatal projection. Thus, interconnected cortical areas shared projection targets in basal ganglia, but this relationship was stronger for the IT-type subset of corticostriatal projections.

### Arborization and targeting of single IT-type and PT-type axons

**Fig. 7:**
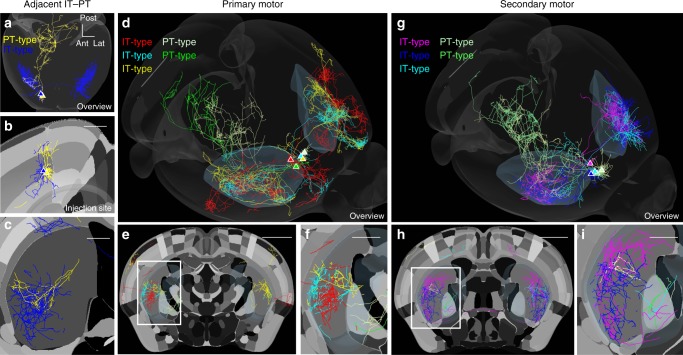
Single neuron reconstructions of IT-type and PT-type neurons in motor areas. **a** Reconstruction of the long-range axonal projections of adjacent PT-type (gold) and IT-type (blue) L5 pyramidal neurons^34^. Projections throughout the whole CNS anterior to medulla are shown. The outline of the reference brain is shown. Scale, 1 mm. **b** Somata are in adjacent in primary motor cortex (M1). In coronal sections (**b**, **c**, **e,** **f**, **h**, **i**), the CCF reference atlas regions are shown in grayscale, with each area or cortical layer for a given cortical area shown in a distinct shade of gray. Scale, 0.5 mm. **c** Corticostriatal projections show similar general topography with differences in arbor size and density. Scale, 0.5 mm. **d** Five adjacent IT-type (red, teal, and gold) and PT-type (green and white) M1 neurons. PT-type neurons shown arborize in thalamus, superior colliculus, pons, and medulla in addition to ipsilateral striatum. The 3D outline of the reference brain is shown, with ipsi- and contralateral striatum highlighted in light gray. **e**, **f** Corticostriatal projections show topography of IT-type and PT-type projections, as well as differences in density, terminal field size, and asymmetry of projections to contralateral striatum. White box in **e** magnified in **f**. Scale, 1 mm (**e**), 0.5 mm (**f**). (**g**–**i**) Five adjacent IT-type (purple, teal, and blue) and PT-type (green and off-white) secondary motor cortex (M2) neurons. Scale, 1 mm (**h**), 0.5 mm (**i**)

Mean projections were based on ~600–900 neurons per injection (IT-type injections 906.9 ± 71.7, PT-type injections 612.1 ± 44.7, mean ± sd). Examination of axonal arbors of single neurons shed light on how variable the projections of each population of pyramidal neurons might be. In the MouseLight project^34^, single axons of IT- and PT-type cells in primary and secondary motor cortex were imaged and registered to the Allen Reference Atlas. Although a limited number of total neurons were available, individual axons extended certain aspects of these findings. IT-type and PT-type neurons in the same area shared a similar corticostriatal topography, though IT-type arbors were more extensive and PT-type arbors were more focal (Fig. 7a-c). Comparison of multiple primary motor cortex (M1) projections confirmed that larger IT-type arbors have more overlap, while more focal PT-type projections were less likely to overlap. From the same area, PT-type axons innervated a subset of the region innervated by IT-type axons (Fig. 7d–f). The overall pattern of IT-type projections differed between M1 and M2 (Fig. 7g–i): M1 axonal projections targeted more focal areas, while, in contrast, individual M2 axons projected more broadly within the striatum, resulting in considerable overlap and only a rough topographic organization. M2 projections were also stronger to contralateral striatum. Individual IT-type neurons in M1 and M2 showed considerable heterogeneity in terms of bilateral projections, with some neurons projecting axons primarily ipsilaterally, some contralaterally, and some bilaterally (cf. IT-type gold vs. red). Considerable variation between individual IT- and PT-type neurons suggested that further subclassification of these cell types is needed.

### Striatum is loosely organized in topographic areas

**Fig. 8:**
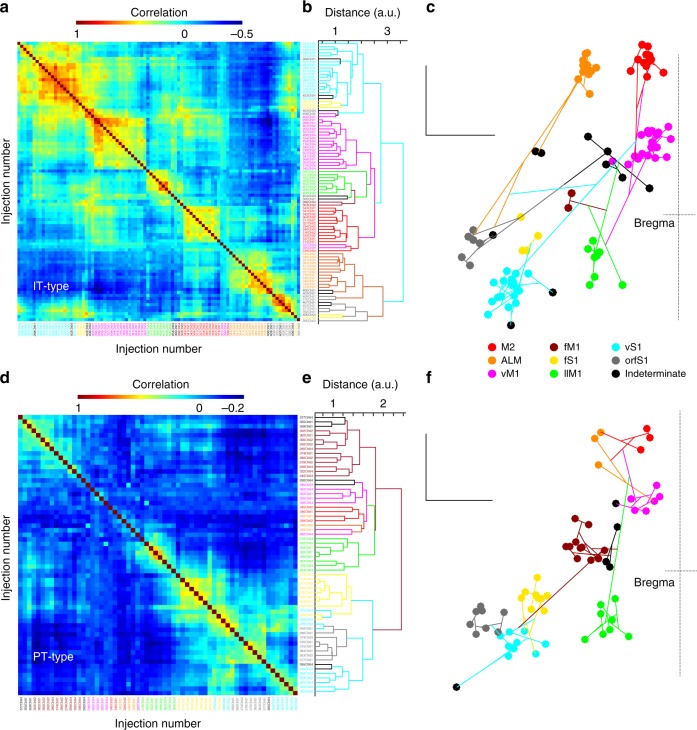
Hierarchical clustering of IT-type and PT-type corticostriatal projections. **a** Pairwise correlation scores for all IT-type projections studied compared for ipsilateral striatal voxels (*N* = 92). Colormap shows high correlation in red and negative correlation in blue. Perfect correlation is along the main diagonal due to comparing an individual case to itself. **b** Each injection case was then hierarchically clustered using differences in the correlation score as the distance measure. Individual injection cases are shows as the free endings of the dendrogram at left and are color coded according to the injection site location cluster to which they were assigned. **c** Using a dorsal view of the brain (with bregma marked at right; scale, 1 mm), the dendrogram from **b** was plotted using the injection site location as the point for the free ending of the tree. Injection site locations displayed as in Fig. 1k. **d**–**f** Pairwise correlation scores and dendrograms for all PT-type projections studied (*N* = 62), plotted as for IT-type projections

IT-type and PT-type projection correlations were used to construct hierarchical relationships between cortical injection sites based on the projections to ipsilateral striatum. Pairwise correlation scores for IT-type outputs were used to construct a dendrogram using Euclidean distance between correlations as the distance measure. Generally, nearby injection sites showed the greatest affinity (Fig. 8a-c). At higher hierarchical levels, most fS1 and vS1 injections clustered together. Motor injections in vM1, fM1, llM1, and M2 also clustered together. Unexpectedly, orfS1 clustered with ALM, suggesting an affinity between lateral sensory and frontal areas in their projections to ipsilateral striatum. Of interest, this affinity also recurred in a similar analysis of corticocortical correlations (Supplementary Fig. 7). In contrast to the IT-type results, using the same methodology to examine PT-type corticostriatal outputs, sensory inputs clustered together, separately from motor and frontal inputs (Fig. 8d-f).

**Fig. 9:**
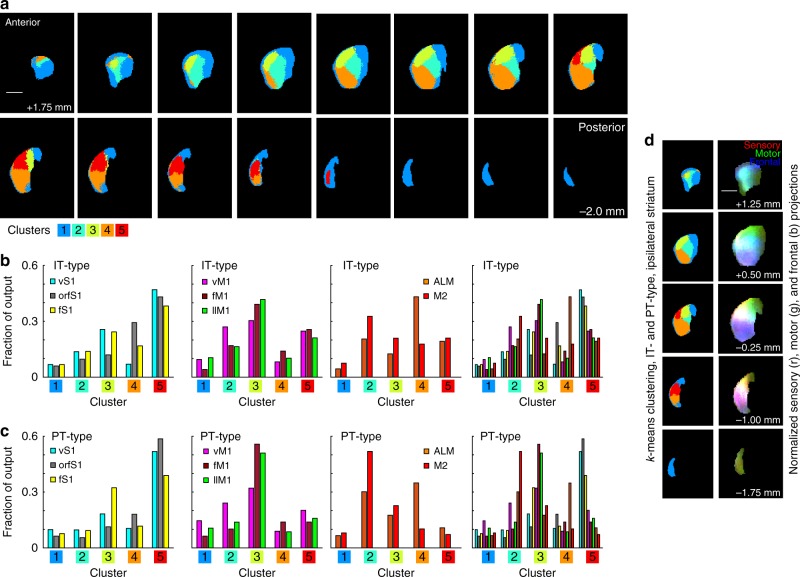
Major divisions of ipsilateral striatum based on sensory and motor cortical projections. **a** *k*-means clustering of striatal pixels based on mean normalized fluorescence intensity from each of the eight injection site clusters for both IT-type and PT-type pyramidal neurons. The striatal clusters are illustrated as five colors (legend, at bottom) in evenly spaced planes every 0.25 mm from anterior (top left) to posterior (bottom right). Scale, 1 mm. **b** The fraction of the output from each of the eight injection site clusters to a given striatal division from IT-type projections. Graphs are divided into sensory (left), motor (center), and frontal (right), with all areas together at far right. **c** The fraction of the output from each of the eight injection site clusters to a given striatal division from PT-type projections, presented as in **b**. **d** A comparison of the striatal divisions based on *k*-means clustering (left) to the pattern of normalized sensory (red), motor (green), and frontal projections (blue), presented as an RGB image (right). Scale, 1 mm

Differences in input contribute to differences in striatal function. Since corticostriatal afferents form a major excitatory input, differences in sensory, motor, and frontal corticostriatal projections could identify functionally distinct striatal regions. Average normalized projection patterns were determined from eight injection clusters for two mouse lines. The normalized projection strength was used to assign ipsilateral striatal voxels into clusters using *k*-means clustering. Five clusters were found based on the peak silhouette value. These were presented in coronal section for the ipsilateral striatum using five colors (Fig. 9a). The fraction of output to each of the clusters is shown for IT-type and PT-type projections (Fig. 9b, c). One cluster (blue) covered the anterior, medial, and posterior edges of the striatum, which were predominantly regions receiving poor output from sensorimotor cortex. The dorsolateral sector included (a) an anterior core region (green) that received substantial M2 and primary motor output, (b) an anterior dorsolateral region (olive) that received strong motor output and some sensory output, and (c) a posterior dorsolateral region (red) that received strong sensory output and some motor output. The ventral and posterior domain received input from ALM and orfS1. This analysis was repeated for IT-type projections alone and PT-type projections alone (Supplementary Fig. 8). Clustering based on IT-type input alone resulted in four clusters, with the anterior and posterior dorsolateral regions that were separable based on both projections combined into a single cluster when PT-type data were excluded. This shift highlighted a difference in the IT-type and PT-type projections: the primary motor projections favored the anterior (olive) dorsolateral cluster, while the primary sensory projections favored the posterior (red) dorsolateral cluster. This difference was more pronounced for PT-type than for IT-type. Thus, differences in PT-type projections identified putative functionally distinct regions of striatum. That these regions were divided by PT-type sensory and motor outputs is also consistent with the earlier dendrogram (Fig. 8). The clustering of IT-type outputs to contralateral striatum was similar to ipsilateral striatum, but not as well defined. Three clusters were sufficient to describe contralateral projections (Supplementary Fig. 8). Consistent with this, the overall correlation coefficients were reduced for these projections (Supplementary Fig. 4). This implied a reduction in the topographic specificity of contralateral striatal projections.

## Discussion

How do the corticostriatal projections of PT-type and IT-type neurons differ? The current data show that corticostriatal projections of interconnected cortical areas replicate their corticocortical connectivity by projecting to shared targets in the striatum, as predicted^2,4^ (Fig. 6). However, this model^2,4^ did not distinguish between cell-type-specific projections. Discriminating between IT-type and PT-type afferents revealed this model best describes IT-type projections. Differences in the corticostriatal topography of projections for specific cell types within a cortical region had not previously been predicted. The basis for this difference is not that the center of mass of corticostriatal fluorescence differs (Fig. 5). Instead, within nearby cortical sites, there is greater heterogeneity in the PT-type projection between animals, as well as between axonal projections of single cells (Fig. 7). Thus, PT-type output is more focal, but less stereotyped in its targeting, compared to IT-type output. Pairwise comparison of PT-type and IT-type injection cases confirms this (Figs 3, 4), as does single-axon data (Fig. 7). These differences have been difficult to appreciate with conventional tracing techniques, though overlapping projections in subcortical targets including thalamus have been effective as a measure of topographic alignment between cortical sites^1^. Fine afferents may be missed in the corticostriatal projection in the Golgi method^35^ and tracers do not distinguish between cell types^2,26^. However, single axon fills in rat have previously demonstrated differences in the degree of corticostriatal terminal arborization between these two cell types^10^. Thus, cell type-specific lines are advantageous for anatomical tracing since the long-range projections of different cell types are organized differently^16,36^.

The relative importance of IT-type and PT-type corticostriatal projections is unclear. Both cell types are significant in rodents, as seen here. PT-type collaterals are also present in primates^37^, but some studies argue are less prominent^14,38^. These neuronal subtypes receive distinct inputs^11^ and convey different classes of information to descending circuits^14^. Thus, these differences may contribute to functional specialization within the striatum. These quantitative assessments of correlation for corticostriatal projections would be difficult to achieve with lower resolution alignment (>100 µm voxels) or the scoring of axons as present or absent (reducing the bit depth of images), which may limit similar studies^2,26,39^. Inclusion of other subtypes of projections, such as L2/3 pyramidal neurons or thalamic inputs^39^, or further subdividing IT-type neurons (as is possible with MouseLight) may reveal more nuanced structure within the corticostriatal projection.

Addressing the functional significance of these anatomical differences is difficult. Focal PT-type output to a smaller subset of striatal neurons might be useful for activation of a targeted subset of the basal ganglia circuitry associated with the specific set of musculature targeted by the brainstem and spinal networks activated by the descending axon of the same PT-type cell. In contrast, IT-type outputs might represent a broader signal to initiate movement or govern its vigor. Thus, the enhanced overlap of IT-type projections from corresponding motor and sensory topographic areas might be more important for more general signals to coordinate movement as opposed to specific signals related to movement initiation of a specific muscle. However, this does not yet explain how each pathway differentially contributes to plasticity during skill acquisition^40^.

The difference in correlation of corticostriatal projections as a function of injection site offset between pairs of nearby primary motor or pairs of primary somatosensory injection cases is remarkable (Fig. 4b, d). In comparing IT-type injections in S1 and M1, the highest correlations are found for nearby injections in S1 (Fig. 4). The steep drop in correlation with increasing distance between injection sites is consistent with a greater topographic specificity in primary somatosensory areas. This is paralleled by functional data, where specific areas of S1 are highly specific for certain body regions such as barrel cortex, where individual barrels are specific for a single whisker^27^. In contrast, microstimulation data suggest that motor representations, while topographically organized, are also generally intermingled^24,25,28,32,33,41,42^. The basis of these topographic differences may derive from the fact that somatosensory cortical areas have a clearly defined input for a given cortical column, such as the primary thalamocortical afferent to layer 4, representing touch of a single finger or whisker^43^. In contrast, primary motor areas have less spatially restricted thalamic^44^ and cortical^29^ inputs. The neurons in these areas may represent a more diverse range of phenomena^45^, ranging from muscles^46,47^ to movements^48^ and behaviors^49^, where body representation alone is not sufficient. It is worth noting that the decrease of correlation with injection site offset is relatively linear instead of stepwise, though smaller steps in the noise are possible. This is consistent with a gradual shift in topographic representation of body regions in striatum instead of discrete segments dedicated exclusively to a single region^2^.

This relationship is also true between sensory and motor injections labeling PT-type neurons, but the overall level of correlation is lower. This was unexpected. These projections, as collaterals of output targeting subcortical targets, were expected to be more precise. The higher correlation of IT-type projections is not due to targeting of a specialized IT-specific striatal region or a substantial offset in the projection zones of the two cell types, as the center of mass of corticostriatal fluorescence for PT- and IT-type projections is similar across the anterior/posterior extent of the striatum (Fig. 5). Instead, quantification of PT-type collaterals showed that these projections have fewer suprathreshold voxels and thus are more spatially limited (Figs 3, 4). Individual axon reconstructions, such as MouseLight data, show that striatal axons of IT neurons are more highly branched than those of PT neurons^10^. Therefore, individual PT-type terminals are more focal (Fig. 7). But these PT-type terminals also show less spatial overlap and higher variability for nearby injection site cases (Fig. 4) or between nearby cells (Fig. 7). This correlation is not simply due to a reduction in the volume of overlap, as comparisons between PT- and IT-type injections in nearby sites showed an increase in overlap volume, but relatively low correlations comparable to PT–PT correlations for the same injection site offset (Fig. 4). Thus, peak correlation is not simply driven by overlap volume.

Although there is strong evidence from primates^4^ and rodents^50^ for convergence of corticostriatal afferents from associated cortical areas, some data^51^ suggests S1 and M1 projections are largely non-overlapping. This result may differ from those presented here if the topographic alignment of the two sites is imprecise (Figs 4, 6). The dual channel recordings presented here (Supplementary Fig. 2) show synaptic convergence of S1 and M1 outputs for all SPNs recorded, demonstrating that integration of topographically aligned sensory and motor signals is a relatively frequent characteristic of striatal neurons.

Contralateral corticostriatal projections of IT-type neurons show reduced correlations compared to ipsilateral axons (Supplementary Fig. 4). Thus, the precision of axonal targeting varies across different collaterals of the same cell type. Since it would be possible to use the same molecular and activity-dependent cues to achieve the same precision in ipsi- and contralateral connections, it will be interesting to learn the functional import of generating a contralateral projection with less spatial precision than the ipsilateral one. On the one hand, longer-range contralateral projections might lose some topographic precision, but how does the animal benefit from a less precise contralateral projection? Such inputs would seemingly degrade the precision of input to contralateral SPNs.

Notably, overall projection density differs across IT-type and PT-type neurons moving from frontal to motor and sensory areas by number of supratheshold voxels (Fig. 5) and mean voxel intensity (Fig. 3c, i). In IT-type injections, frontal projections provided the densest striatal afferents. In contrast, for PT-type injections, frontal injections were by contrast the weakest (Figs 3, 4). Thus, PT-type projections contribute to a relatively higher proportion of the total corticostriatal output from sensory areas. This difference is useful in subdividing the striatum into sectors, where including both PT- and IT-type projection data helps differentiate anterior and posterior dorsolateral striatal areas specialized for motor and sensory input, respectively (Fig. 9, clusters 3 and 5) which merge when IT-type only output is considered (Supplementary Fig. 8a). The precision of the striatal subdivisions presented is stronger in dorsolateral areas than ventrally, where corticostriatal afferents originate outside sensorimotor areas and were not sampled. However, even with dense sampling, it was not straightforward to identify clusters exclusive to specific body parts without overlap.

Several sources may limit the ability to measure stereotypy of corticostriatal projections. Relatively compact versus scattered injection sites did not show a large variation in corticostriatal correlations, suggesting that injection site size did not play a major role in variation between injections. Thus, animal-to-animal variation instead of injection variability may play a larger role in limiting the peak correlation. Other limitations include the spatial resolution of the alignment (~50–70 µm) and voxel size, which could reduce correlations by spatial averaging. Using higher resolution aligned images (10 × 10 × 10 µm voxels) did not alter the linkages between injection sites (data not shown). That peak correlations are close to 0.6 suggests that animal-to-animal variation sets an upper limit on comparisons across brains. That peak correlations are not closer to 1.0 quantified the substantial inter-case variability and underscores the relevance of studying injections across cases in different animals instead of using a single injection case to assess typical projection targets in striatum.

Although frontal areas, such as ALM and M2, might be organized differently than sensory and primary motor cortex, it was interesting that IT-type projections from lateral regions of frontal cortex (ALM) projected to striatum similarly to those originating from orofacial regions of S1 anterior and lateral to barrel cortex. Of note, the corticocortical collaterals of ALM also projected posteriorly toward lateral regions of motor and somatosensory cortex. This was reciprocated by projections from orfS1 to ALM. Thus, ALM’s corticocortical connectivity suggested a basis for corticostriatal overlap with orfS1 projections. Coincidentally, ALM has been identified as a low-threshold region for evoking tongue movement in rodents^24,52,53^. Based on this connectivity pattern, ALM and orfS1 are connected in a manner reminiscent of primary motor and sensory regions, as previously suggested^26^. ALM has also been implicated in more traditional frontal cortex functions such as motor planning in mice^54,55^.

A comprehensive study of differences in cortical cell type (IT or PT type) output to distinct striatal populations was not possible from all cortical areas to the range of striatal neuron populations, including direct and indirect SPNs as well as striatal interneuron populations. Targeting of afferents to distinct striatal compartments such as patch and matrix may also differ between different cortical areas, though average sensorimotor populations target both patch and matrix neurons in similar proportions^56^. Afferents from both IT- and PT-type cells form connections to direct and indirect-pathway SPNs^57^. It not yet possible to evaluate, however, whether there is a bias in targeting from either PT- or IT-type output, as has been proposed^58,59^, because of quantitative limitations in circuit mapping methods. Retrograde tracing with transgenic rabies suggests that sensory and motor inputs preferentially excite direct and indirect-pathway SPNs, respectively^60^, suggesting specific postsynaptic targeting of afferents is possible in striatum. Physiological data (Supplementary Fig. 2) show that motor and sensory corticostriatal afferents converged on single SPNs (Supplementary Fig. 2), but did not quantitatively distinguish between the cell types targeted.

Layer-specific Cre-driver lines such as Tlx3_PL56 and Sim1_KJ18 lines may not collectively label all L5 pyramidal neurons. For example, in the L5 mouse line Rbp4_KL100, some IT-type and PT-type neurons are labeled, but the overall labeling density leaves many cells of both classes unlabeled^18^. The density of PT-type and IT-type neurons in Sim1_KJ18 and Tlx3_PL56 lines varies over cortical areas, which suggests that some neurons may be missed in different regions. There may be underappreciated heterogeneity within these two L5 populations, such as different subtypes of IT neurons for different targets^61^. Furthermore, in ALM injections of frontal cortex in Sim1_KJ18 mice, some contralateral axonal projections are present. In other areas, such as midline cortical areas where lamination is less pronounced, transgenic reporters for these lines suggest changes in Cre expression, resulting in reduced Tlx3_PL56 and Sim1_KJ18 labeling^18^. Thus, use of transgenic approaches to target specific cell types is limited to the brain regions where these cell types are well characterized.

The corticostriatal projection formed by two populations of L5 pyramidal neurons conveys distinct functional information with distinct striatal targeting. IT-type neurons in sensory and motor areas target topographically organized domains of striatum and also overlap substantially with other cortical areas with which they are reciprocally connected. PT-type neurons, in contrast, show less overlap with reciprocally connected cortical areas. This difference suggests that the measured degree of topographic organization depends in part on the cell type considered. As these cell types convey distinct information to striatum, it remains to be determined what purpose this differential targeting serves.

## Methods

### Injections

All breeding, surgical, and experimental procedures conformed to National Institutes of Health guidelines for mice and were approved by the Institutional Animal Care and Use Committees of University of Pittsburgh and Janelia Research Campus. Mice from four GENSAT BAC Cre-recombinase driver lines (Sepw1_NP39, *N* = 7; Tlx3_PL56, *N* = 33; Sim1_KJ18, *N* = 22; and Ntsr1_GN220, *N* = 5)^18^ were used to trace the projections of four populations of cortical pyramidal neurons. Mice of both sexes were injected at postnatal day P37.0 ± 1.7 (mean ± se) and sacrificed after 2–3 weeks of expression. All stereotaxic injections targeted the same hemisphere. Injection sites covered a range of topographic locations in primary somatosensory cortex and corresponding areas of motor and frontal cortex^24–26^. 30 nL per injection site of AAV-flex-XFPs were injected using a custom positive displacement injector via a pulled borosilicate glass micropipette^29^. The generic AAV-flex-XFP refers to several tracing viruses used, including AAV2/1-CAG-flex-EGFP, AAV2/1-CAG-flex-tdTomato, and the GFP- and mRuby2-based spaghetti monster fluorescent proteins (smFPs) smFP-FLAG, smFP-Myc, smFP-V5, smFP-HA, Ruby2-FLAG, and Ruby2-OLLAS (Table 1)^19^. Injections were made into cortex (at 300–1100 µm depth). For injections into L5 and L6, virus was injected at two depths. Laminar specificity was achieved by Cre-recombinase instead of injection depth. Typically, three sites were injected per mouse. In some cases, fewer channels were quantified if expression was not usable in a given channel due to weak expression or marked spread of the virus away from the injection site.

### Histology, staining, and imaging

Mice were transcardially perfused with 4% paraformaldehyde in phosphate-buffered saline and postfixed overnight. Brains were then transferred to 20% sucrose in PBS for storage. Brains were sectioned at 80 µm and signal was immunoamplified. 1:100 dilution of Neurotrace Blue was used as a structural marker^20^. Sections were then imaged using Neurolucida (v2017, MBF Bioscience, Williston, VT) on a Zeiss Axioimager (Zeiss, Oberkochen, Germany) equipped with 10x objective, Ludl motorized stage and a Hamamatsu Orca Flash 4.0 camera (Hamamatsu, Hamamatsu City, Japan). Each section was comprised of an average of ~100–200 image stacks collected in 10 µm steps. A single 3D image was first generated then a deeper field-of-view was achieved by collapsing images to a single plane using a DeepFocus algorithm^18,20^ (Supplementary Fig. 1b-e) implemented in Neurolucida. Original images are available at: http://gerfenc.biolucida.net/link?l=Jl1tV7

### Whole-brain reconstruction, annotation, and registration

Tiled images were aligned to a standard coordinate system using BrainMaker software (MBF Bioscience, Williston, VT). Resulting serially reconstructed brains contained 10 µm isotropic voxels (782 × 1086 × 1242) and were registered to the annotated Allen Mouse Common Coordinate Framework (CCF), Version 3 (http://connectivity.brain-map.org)^15–17^. All brains were registered to this framework using Neurotrace Blue images as the structural marker and a two-stage registration process. The first stage constructed an average reference space that provides a representation of the average appearance of brains that have undergone histological sectioning, mounting, and staining specific to this study and in the same image channel (i.e., Neurotrace Blue). Registration of individual brains to this average reference space was found to be more robust than direct multimodal registration to the Allen CCF reference image.

The average reference image was constructed from 78 individual 3D brains in a manner similar to the Allen CCF, which incorporates 1675 individual brains with cytoarchitecture visualized with 2-photon auto-fluorescence^17^. In this study, the counterstain (Neurotrace Blue) channel for each individual brain was registered to a reference template, initialized as one of the individual brains resampled with a uniform voxel spacing. Multiple resolution registration optimized the 12 parameters of a 3D affine transform to minimize a normalized correlation metric between each brain and the template image. The reference template was then updated by resampling all individual brains with their respective affine transforms and computing a voxel-wise weighted average. Voxels that received a small number of contributions were discarded to correct for some tissue damage present in individual brains. A second pass registered each individual brain to the new template, updating the individual transforms. This process repeated until the template image stabilized.

The second stage involved registering the average reference image to the Allen CCF. 300 unique landmark points were identified in the average reference image and corresponding points in the Allen CCF 2-photon reference image. The positions of the landmark correspondences were used to construct a nonlinear transform that models deformation of a uniform mesh grid with B-splines. This transform was used to resample the Allen CCF annotation volume in the average reference image using nearest neighbor interpolation. The result, an average reference image and its spatially aligned annotation volume, constitutes the average reference atlas. The counterstain channels of individual brains in this study were registered with the average reference space by adjusting parameters of a 3D affine and 3D nonlinear B-spline transform to minimize a normalized correlation metric. Some but not all individual brains contributed to the average reference space. Measurement of alignment precision showed this was accurate to ~50–70 µm (Supplementary Fig. 1l-y). Comparable studies use alignment methodologies with less precision (~100 µm), larger voxels (100–150 µm per side)^39^ or images reduced from 8-bit to 2-bit (“dense/strong”, “moderate”, “diffuse/light”, etc.)^2,26^.

The recovered transform was used to map the locations of fluorescence and cell soma locations detected on fluorescent tracer channels. For quantification of injection site location, tiled images were imported into Neurolucida software (MBF Bioscience, Williston, VT) and soma locations were annotated using automated object detection with manual supervision. Nearest neighbor interpolation of the average reference space volume at the mapped positions provided the anatomical region assignment for each cell. Coordinates of the CCF for structures of interest (such as striatum) were used to identify voxels for quantification. These were divided into left and right hemispheres to distinguish between structures ipsilateral and contralateral to the injection site.

### Data analysis

Aligned brain images were downsampled to 50 µm isotropic voxels (156 × 217 × 248) using custom routines in FIJI software^62^. The annotated Allen Mouse CCF was also used at 10 µm and downsampled to 50 µm. The annotation was used to assign voxels to a given brain region (ipsilateral or contralateral striatum, for example). Both 10 µm and 50 µm images were converted from tifs into.mat files in Matlab (Mathworks, Natick, MA) for analysis with custom routines. Soma locations were similarly imported to Matlab.

### MouseLight reconstructions

Individual neurons are reconstructed using fast volumetric serial two-photon (STP) tomography^34^ with voxels of 0.1 µm^3^, scanning a cleared mouse brain in ~1 week. Low numbers of individual neurons were labeled using high titer ( > 10E12 GC/ml) AAV 2/1 CAG-Flex-eGFP with dilute (1:45,000) AAV 2/1 Syn-iCre injected into cortex. Images were tiled in smaller 3D stacks (tiles in XY) and the sample translated using a mechanical stage. Scanning in the Z-direction was achieved with a piezoelectric to move the objective. Tissue was sectioned with a custom Leica 1200 S integrated with the imaging system. Both green and red channels are acquired. Neurons were manually reconstructed. Images were registered using custom Matlab scripts. Tiles were resampled into a common coordinate space with affine transforms determined during registration.

## Electronic supplementary material

Supplementary Information

## Data Availability

The data that support the findings of this study are available from the corresponding authors upon request. Aligned images in 10 and 50 μm voxels for all brains, cell soma locations, and the corresponding masks used to identify brain regions (striatum, for example) are available on request. Custom Matlab code for data analysis is available on request. Original images of whole brains are freely available online at: http://gerfenc.biolucida.net/link?l=Jl1tV7.
