# Author Correction: Topographic precision in sensory and motor corticostriatal projections varies across cell type and cortical area

**DOI:** 10.1038/s41467-018-06928-1

**Published:** 2018-10-12

**Authors:** Bryan M. Hooks, Andrew E. Papale, Ronald F. Paletzki, Muhammad W. Feroze, Brian S. Eastwood, Jonathan J. Couey, Johan Winnubst, Jayaram Chandrashekar, Charles R. Gerfen

**Affiliations:** 1 Department of Neurobiology, University of Pittsburgh School of Medicine, Pittsburgh, PA USA; 2 Laboratory of Systems Neuroscience, NIMH, Bethesda, MD USA; 3 MBF Bioscience, Williston, VT USA; 4 Janelia Research Campus, Ashburn, VA USA

Correction to: *Nature Communications* ; 10.1038/s41467-018-05780-7; published online 3 Sep 2018.

In the original version of this Article, support provided during initiation of the project was not fully acknowledged. The PDF and HTML versions of the Article have now been corrected to include support from Karel Svoboda, members of the Svoboda lab, and members of Janelia’s Vivarium staff.

