# Supplementary material for: Topographic precision in sensory and motor corticostriatal projections varies across cell type and cortical area
Source: Nat Commun. 2018 Sep 3;9:3549. doi: 10.1038/s41467-018-05780-7 (PMC6120881; doi:10.1038/s41467-018-05780-7)
Supplement: Supplementary file 1 — Supplementary Information [file 41467_2018_5780_MOESM1_ESM.pdf]

Supplementary Figures

Topographic precision in sensory and motor corticostriatal projections varies across cell type and cortical area

Hooks et al.

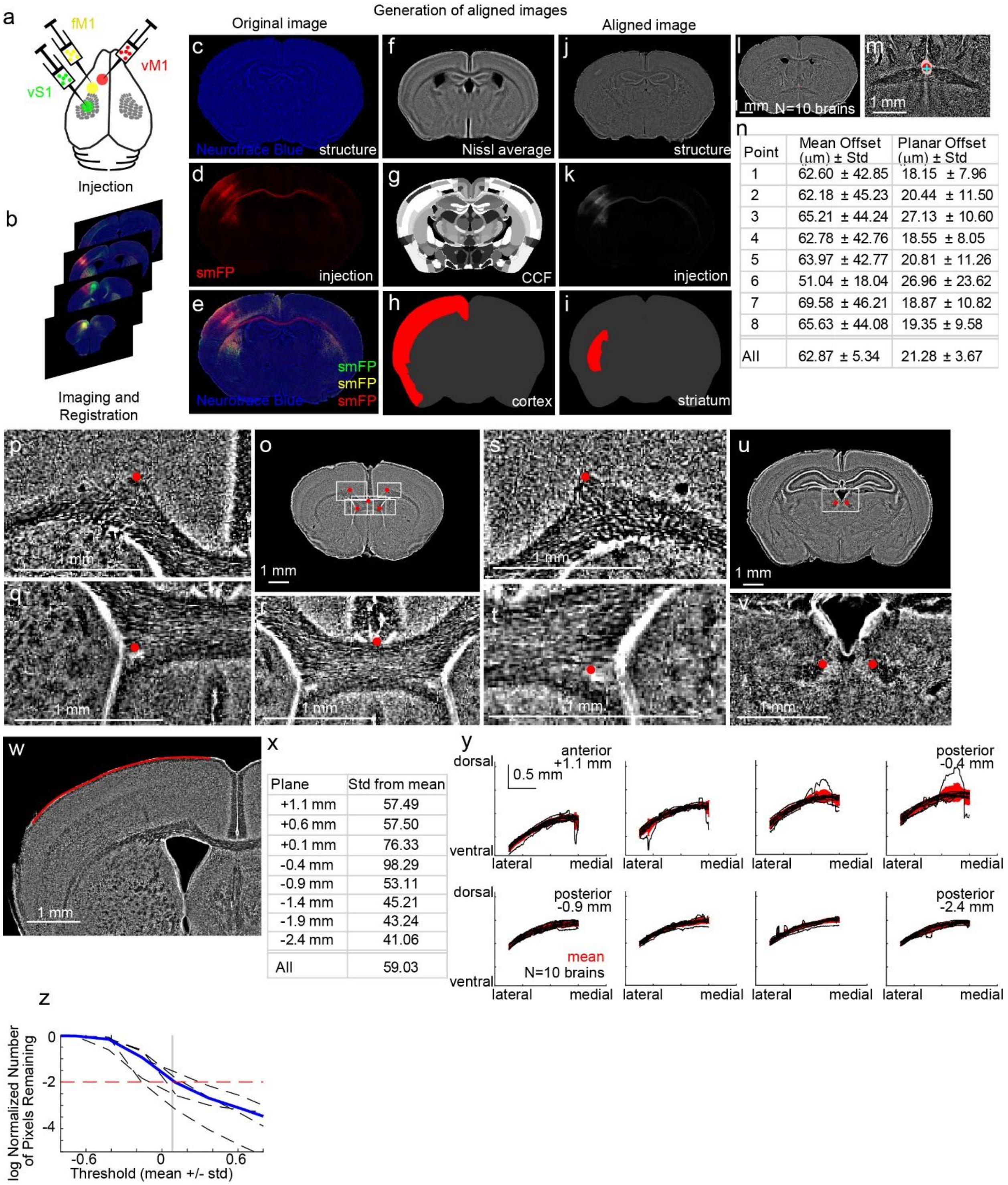

a
fM1
vM1
vS1
Injection
b
Imaging and
Registration
Generation of aligned images
Original image
Aligned image
c
Neurotrace Blue
structure
d
smFP
injection
e
smFP
smFP
Neurotrace Blue
smFP
f
Nissl average
g
CCF
h
cortex
i
striatum
j
structure
k
injection
l
1 mm
N=10 brains
m
1 mm
n
Point | Mean Offset (μm) ± Std | Planar Offset (μm) ± Std
1 | 62.60 ± 42.85 | 18.15 ± 7.96
2 | 62.18 ± 45.23 | 20.44 ± 11.50
3 | 65.21 ± 44.24 | 27.13 ± 10.60
4 | 62.78 ± 42.76 | 18.55 ± 8.05
5 | 63.97 ± 42.77 | 20.81 ± 11.26
6 | 51.04 ± 18.04 | 26.96 ± 23.62
7 | 69.58 ± 46.21 | 18.87 ± 10.82
8 | 65.63 ± 44.08 | 19.35 ± 9.58
All | 62.87 ± 5.34 | 21.28 ± 3.67
p
1 mm
o
1 mm
s
1 mm
u
1 mm
q
1 mm
r
1 mm
t
1 mm
v
1 mm
w
1 mm
x
Plane | Std from mean
+1.1 mm | 57.49
+0.6 mm | 57.50
+0.1 mm | 76.33
-0.4 mm | 98.29
-0.9 mm | 53.11
-1.4 mm | 45.21
-1.9 mm | 43.24
-2.4 mm | 41.06
All | 59.03
y
dorsal
ventral
anterior
+1.1 mm
0.5 mm
posterior
-0.4 mm
posterior
-0.9 mm
posterior
-2.4 mm
mean
N=10 brains
lateral
medial
z
log Normalized Number of Pixels Remaining
Threshold (mean +/- std)
0
-2
-4
-0.6
0
0.6

Supplementary Figure 1 | Generation of Aligned Sensory, Motor, and Frontal Injections
(a) Stereotaxic injection of AAV-flex-smFPs were made into Cre-driver lines labeling cortical pyramidal neurons. Colors (red, yellow, and green) indicate injection sites of AAV plotted on a drawing of dorsal cortex. (b) Brains were sectioned coronally at 80 µm spacing, imaged on a Zeiss Axioimager, and tiled in Neurolucida. (c-e) Original images from a sample plane of a 10 µm brain include Neurotrace Blue (c) to label gross morphology as well as (d) the red fluorescence channel (tdtomato, injected in vS1 of a Tlx3_PL56 mouse). In (e), fluorescence of all three tracer channels is shown. (f) Coronal plane from the Nissl average brain. (g) In the Allen CCF, different brain regions are illustrated in different shades of gray. Brain regions are used to create masks to assign voxels to different structures. (h, i) Mask for a plane of ipsilateral cortex and ipsilateral striatum. Cortex mask is based on brain regions for L1-L5 of all cortical regions. L6 is omitted from analysis to minimize contributions from fibers of passage. (j and k) Individual brains were aligned to the Nissl average with BrainMaker software. Aligned images of Neurotrace Blue structure (j) and the red fluorescence channel (k). (l) Quality of alignment was verified across the Neurotrace Blue channel of 10 example brains. A set of 8 anatomical points was marked in the aligned structural channel, including the anterior commissure (m), the anterior extent of the corpus callosum (o, r), and the point at which the fasciculus retroflexus leaves the 3rd ventricle (u,v). Additional points included the dorsal peak of ipsi- and contralateral white matter coplanar with the corpus callosum, and the midpoint of where the lateral ventricles intersect with white matter. The offset among these coordinates were compared (n) and found to vary by ~62.9 µm ± 5.3 µm. Due to sectioning at 80 µm planes, the offset within the plane (ignoring anterior/posterior offset) was even more precise (planar offset column). (w-y) As a secondary measure of alignment quality, a curve along the dorsal surface of cortex for each of N=10 brains was compared to the mean cortical surface across a range of anterior/posterior positions (+1.1 mm anterior to bregma to -2.4 mm posterior). Individual brains in black and mean surface in red for each plane (y). Red traces include +/- sd. Except for one case with significant slicing artifact (top right), curves differed by ~59.0 µm. (z) Most structures in aligned 50 µm voxel brains contained large numbers of low fluorescence voxels where no axonal projections were expected to be present. Including background fluorescence in correlation analysis dilutes signal in the data. Thus, images were thresholded images to eliminate background voxels. Four structures not expected to contain axonal fluorescence were selected for evaluation (dotted lines): contralateral cortex (in Sim1_KJ18), contralateral striatum (in Sim1_KJ18), bilateral hippocampus (in Tlx3_PL56), and ipsilateral thalamus (in Tlx3_PL56). Mean fluorescence for a given channel was determined and the fraction of suprathreshold voxels remaining for thresholds set near the mean ± 1 standard deviation (STD) was assessed. Variation of threshold in units of 0.1 STD showed differences in the fraction of remaining voxels. The mean is shown in blue. At mean + 0.1 STD (gray line), <99% of background voxels remain. This threshold was used across injections. The data is plotted on a $\log_{10}$ scale. The brain image in panel (a) is adapted with permission from refs. 29-30.

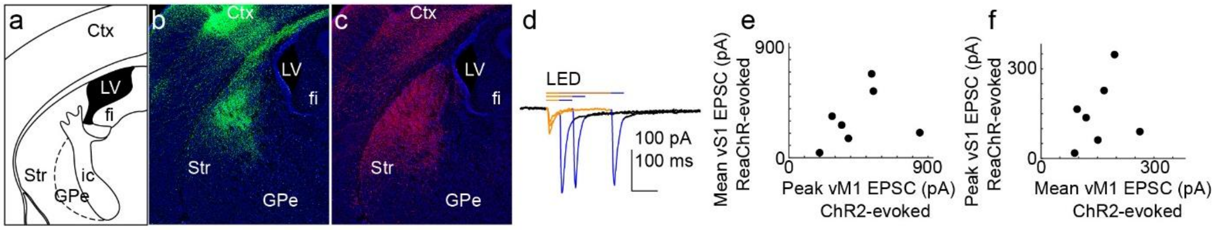

Supplementary Figure 2 | Overlapping sensory and motor projections to striatum converge upon single striatal projection neurons in striatum

(a-c) Projections from vS1 (green, b) and vM1 (red, c) converge in dorsolateral striatum. vS1 axons expressed ReaChR-mCitrine (green) and vM1 axons expressed ChR2-tdtomato. Ctx, cortex; fi, fimbria of the hippocampus; GPe, external globus pallidus; ic, internal capsule; LV, lateral ventricle; Str, striatum. (d) Example recording from a single medium spiny neuron. Traces represent the average of three traces and were recorded under each of three stimulus paradigms. LED indicates when orange (590 nm) LED is on for 50 ms, 100ms, or 250 ms, followed by 50 ms blue (470 nm) LED pulse. Traces colored to indicate responses during corresponding illumination. Scale bar, 100pA and 100 ms. (e and f) Peak and mean evoked EPSC amplitude from vS1 axons and vM1 axons.

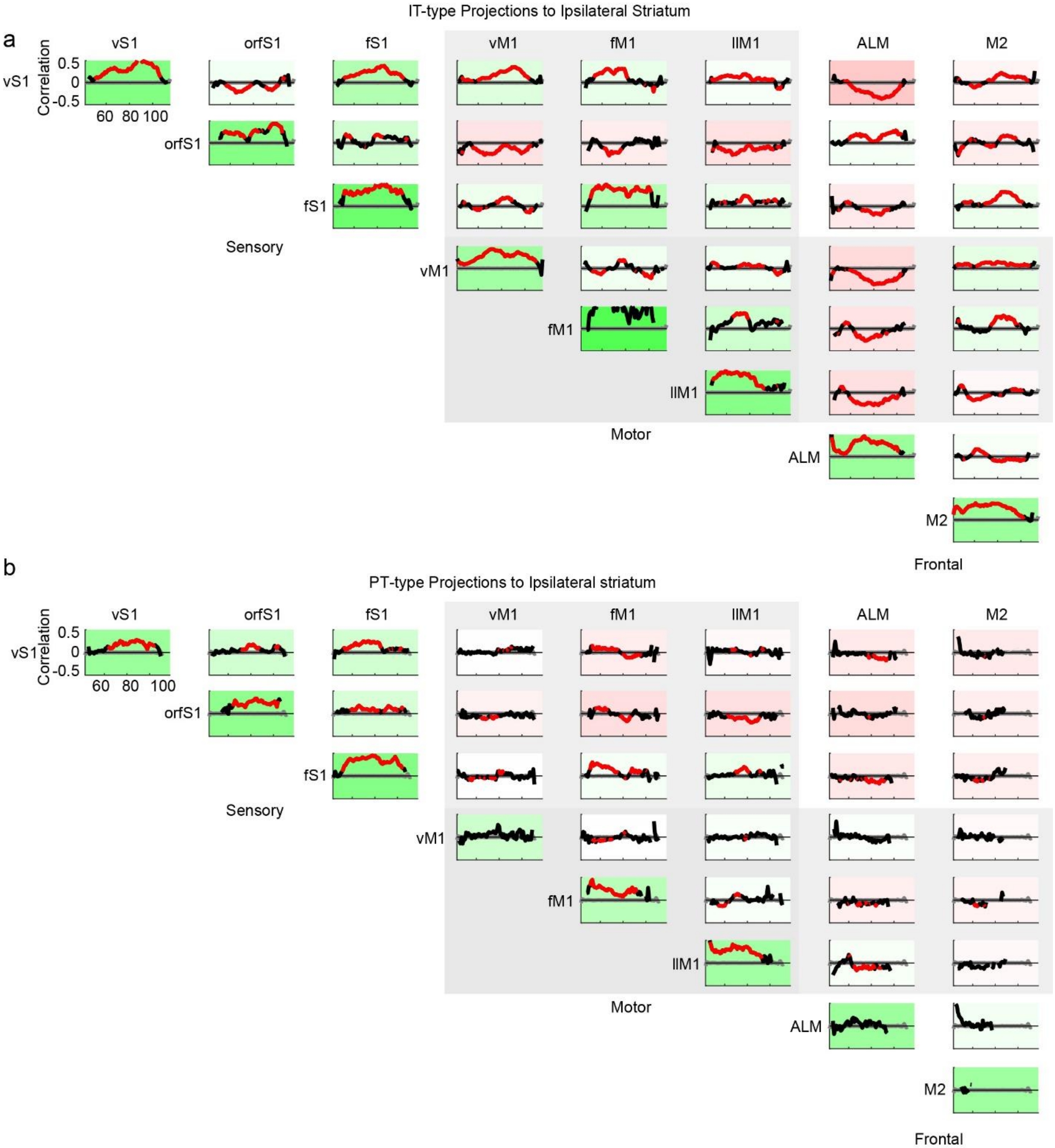

Supplementary Figure 3 | Correlation of corticostriatal projections from IT-type and PT-type neurons for sensory, motor, and frontal injections.

Correlation across the anterior/posterior range of ipsilateral striatum for each of eight injection clusters. (a) Top panels, IT-type; (b) bottom panels, PT-type. Injection site clusters form the rows and columns of the matrix. Correlation is plotted in black, with red points indicating significant differences to shuffled data ($p<0.001$, rank sum test, comparisons for >100 voxels). Mean correlation across all striatal voxels is represented by green (positive correlation) and red (negative correlation) background, with the intensity of color proportional to correlation from 0 (white) to 1 (green) or -1 (red).

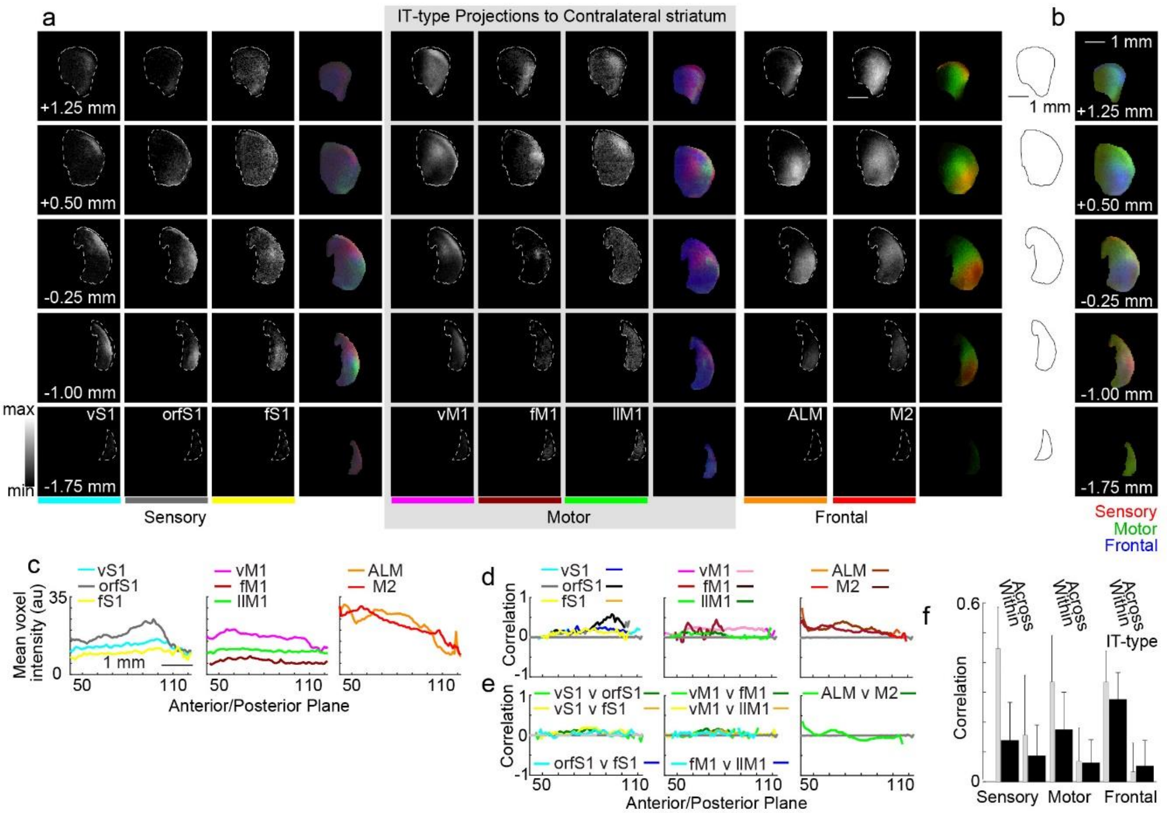

a
IT-type Projections to Contralateral striatum
b
1 mm
+1.25 mm
+0.50 mm
-0.25 mm
-1.00 mm
-1.75 mm
max
min
vS1
orfS1
fS1
vM1
fM1
llM1
ALM
M2
Sensory
Motor
Frontal
c
vS1
orfS1
fS1
Mean voxel intensity (au)
35
0
1 mm
50
110
vM1
fM1
llM1
ALM
M2
Anterior/Posterior Plane
d
Correlation
1
0
-1
e
vS1 v orfS1
vS1 v fS1
orfS1 v fS1
vM1 v fM1
vM1 v llM1
fM1 v llM1
ALM v M2
Anterior/Posterior Plane
f
0.6
0
Across
Within
IT-type
Correlation
Sensory
Motor
Frontal

Supplementary Figure 4 | Topography of sensory, motor, and frontal contralateral corticostriatal projections from IT-type neurons

(a) Coronal images of average contralateral corticostriatal projections from IT-type neurons. Images shown at five anterior/posterior sections from anterior (+) 1.25 mm to bregma to posterior (-) 1.75 mm to bregma. Outline of the contralateral striatum is superimposed as a dashed white line. Scale bar is 1 mm (top panel). Voxels are 50 µm. The eight injection site clusters are organized into sensory (vS1, orfS1, and fS1), motor (vM1, fM1, llM1), and frontal (ALM and M2) modalities. Black and white images show average normalized projections for a given injection site cluster. These are color coded and presented together at the right to show within-modality topography. vS1 projections in red are generally more dorsal, orfS1 in green are generally more ventral, and fS1 are more anterior. (b) Average normalized sensory (red), motor (green), and frontal (blue) projections are shown to illustrate topography across modalities. (c) Quantification of mean voxel intensity across the anterior/posterior extent of ipsilateral striatum. Scale bar 1 mm; each plane is 50 µm. Each injection site cluster is color coded (after Fig. 1). (d) Within cluster comparisons show high correlation for nearby injections in the same cluster. Plot shows mean correlation for a given vS1 injection compared to other vS1 injections. Two colors are used (left, teal; right, blue) with the blue color indicating locations along the anterior/posterior axis where correlation coefficient is significantly different from shuffled data ($p<0.001$, rank sum test). Legend for all comparisons shows two colors for each injection site cluster (right color, significant differences). Similar comparison performed for all eight clusters. Comparisons made in planes for injections where both share >100 suprathreshold voxels. (e) Across cluster comparisons compare injections within the same modality. For sensory clusters, vS1 injections are compared to orfS1(green) and fS1 (yellow), and orfS1 injections are compared to fS1 (blue). Across cluster comparisons are also compared for motor (center) and frontal (right) injections. (f) Mean correlations within (such as vS1-vS1) and across (such as vS1-orfS1) contralateral corticostriatal projections from IT-type pyramidal neurons. Contralateral correlations shown in black; corresponding ipsilateral correlations (Fig. 3) shown in gray.

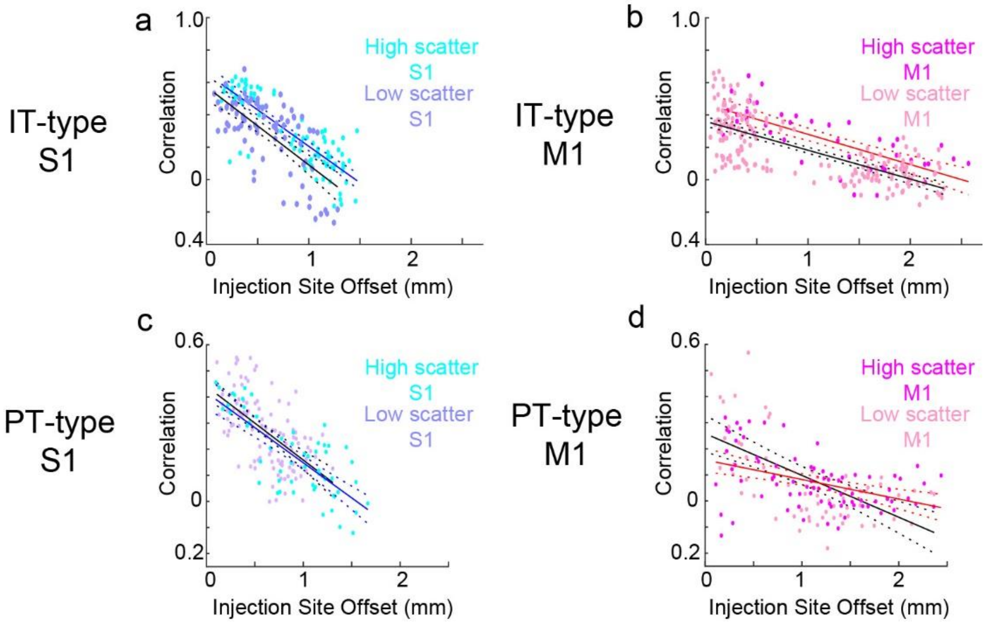

Supplementary Figure 5 | Effect of injection site scatter on correlation
The distance from the center of mass of the injection site for the coordinates of each labeled soma from an injection was computed. The mean distance from the injection site center was used as a measure of injection site scatter and divided injections in each population (S1 and M1) into two groups: those with scatter above ("high scatter") and below ("low scatter") the mean. Pairwise correlation within either the high scatter or low scatter groups were computed and plotted as before. S1 injections labeling IT-type neurons (a) and M1 injections labeling IT-type neurons (b) are presented at top. Results for PT-type neurons in S1 (c) and M1 (d) are plotted below.

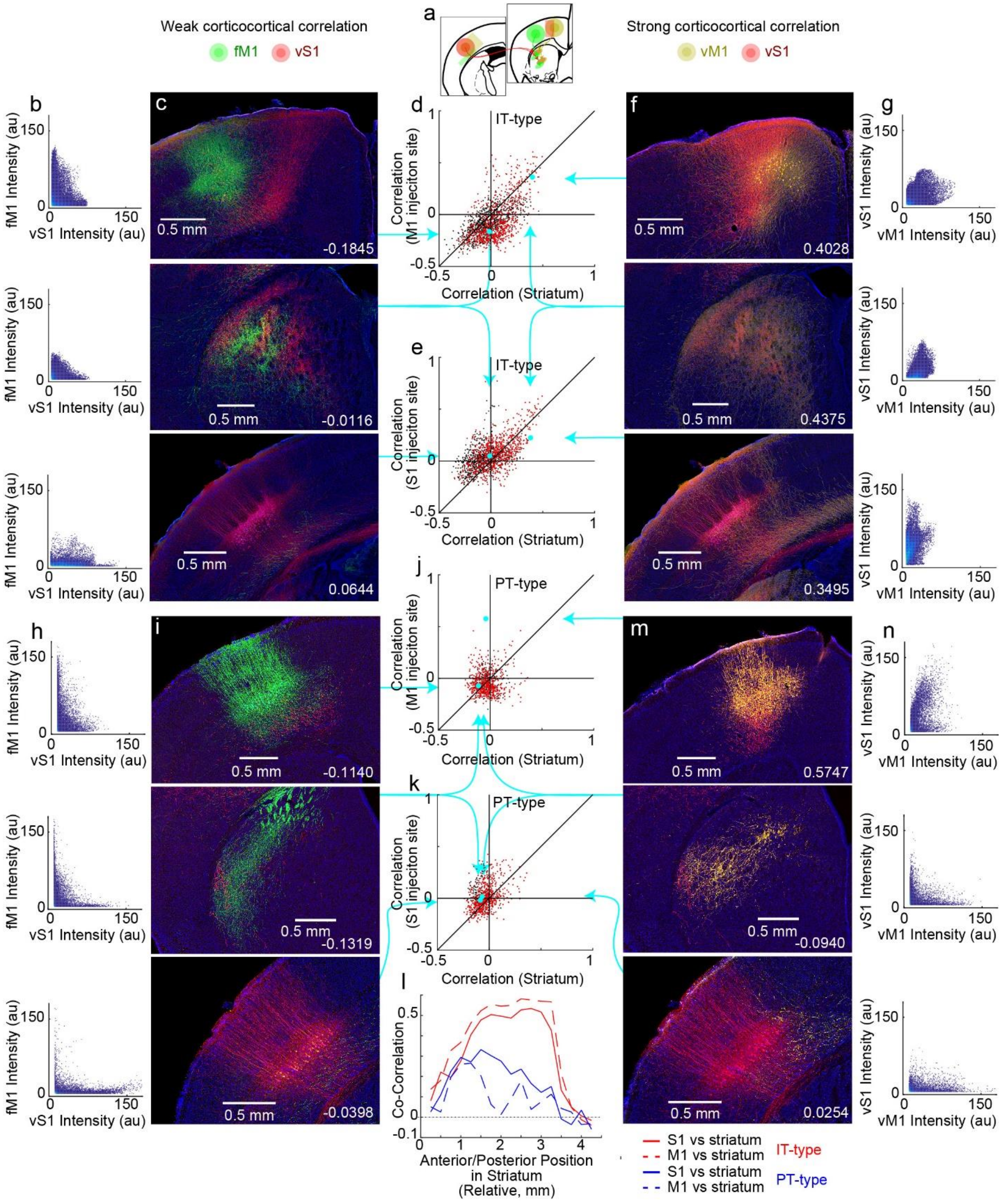

a
Weak corticocortical correlation
fM1
vS1
Strong corticocortical correlation
vM1
vS1
b
c
d
e
f
g
h
i
j
k
l
m
n
IT-type
PT-type
Correlation (M1 injeciton site)
Correlation (S1 injeciton site)
Correlation (Striatum)
fM1 Intensity (au)
vS1 Intensity (au)
vM1 Intensity (au)
0.5 mm
-0.1845
-0.0116
0.0644
-0.1140
-0.1319
-0.0398
0.4028
0.4375
0.3495
0.5747
-0.0940
0.0254
Co-Correlation
Anterior/Posterior Position in Striatum (Relative, mm)
S1 vs striatum
M1 vs striatum
S1 vs striatum
M1 vs striatum
IT-type
PT-type

Supplementary Figure 6 | Corticostriatal projections map the organization of corticocortical connectivity in IT-type but not PT-type projections

(a) Sensory and motor cortex injections make reciprocal intracortical projections between somatotopically related areas. (b, c, f, g) IT-type injection examples shown contrast a pair of strongly connected cortical areas (red vS1 and yellow vM1 injections) with a non-somatotopically aligned area (green fM1 injection). vS1 axons (red) overlap poorly with fM1 neurons (green). These are poorly correlated in both injection sites (-0.1845 and 0.0644; b, c top and bottom) as well as the striatum (-0.0116; b, c middle). In contrast, vS1 axons (red) overlap well with vM1 neurons (yellow) and are strongly correlated in both injection sites (0.4028 and 0.3495; f, g top and bottom) as well as the striatum (0.4375; f, g middle). (d,e) Scatterplot of co-correlations of corticocortical connectivity (using injection site overlap) and corticostriatal connectivity for IT-type projections. Each individual point represents the corticostriatal correlations (x-axis) and injection site correlation (y-axis) for a single pair of injections with corticocortical correlation computed at either M1 (d) or S1 injection sites (e). Red points on the scatterplot compare sensory and motor injections. Black points add comparisons to frontal areas (M2 and ALM). Teal arrows and points indicate specific points corresponding to the example injections shown. (h, i, m,n) PT-type injection examples shown contrast a pair of strongly connected cortical areas (red vS1 and yellow vM1 injections) with a non-somatotopically aligned area (green fM1 injection). (j,k) Scatterplot of co-correlations of corticocortical connectivity and corticostriatal connectivity for PT-type projections. (l) Co-correlations of corticocortical connectivity and corticostriatal connectivity are re-assessed, with corticostriatal correlations (y-axis) calculated using subsets of striatal voxels along the anterior/posterior axis in 250 µm segments (x-axis, in mm). Co-correlation is plotted for IT-type (red) and PT-type (blue) injections.

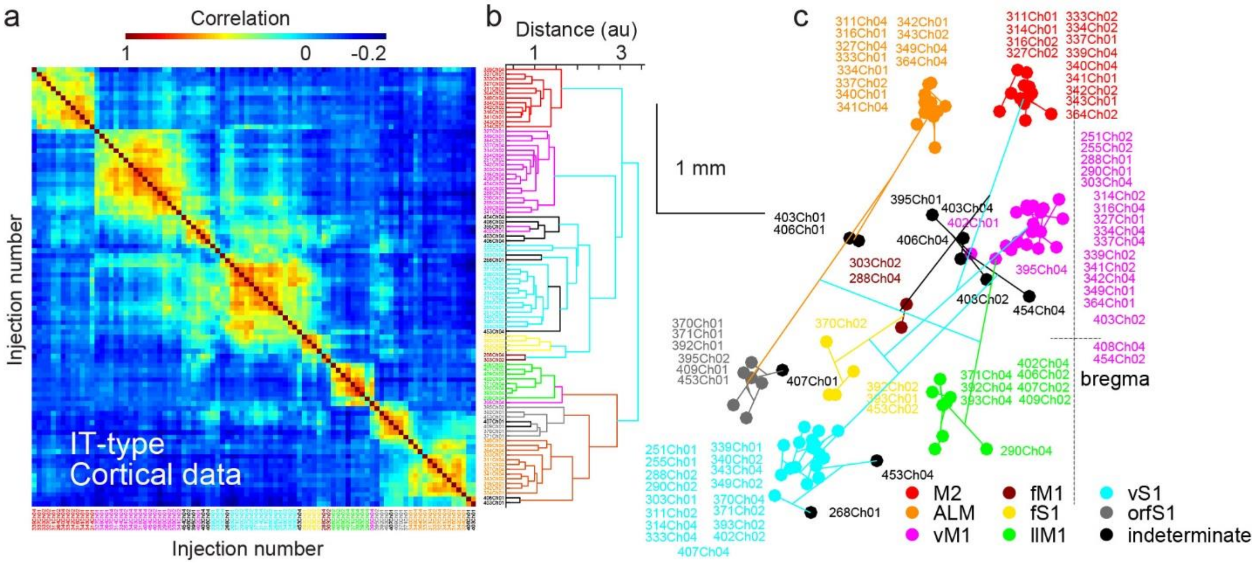

Supplementary Figure 7 | Hierarchical clustering links IT-type sensory and motor projections in cortex

(a) Pairwise correlation scores for cortical projections of all IT-type projections studied (N=92). High correlation, red (with perfect correlation along the main diagonal). Negative correlation, blue. (b) Injections were hierarchically clustered using correlation score as the distance measure. Individual injections at the tips of the dendrogram were color-coded according to the injection site location cluster to which they were assigned. (c) Using a dorsal view of the brain (with bregma marked at right; scale bars, 1 mm), the dendrogram from (b) was plotted using the center of mass of the injection site as the point for the tip of the tree.

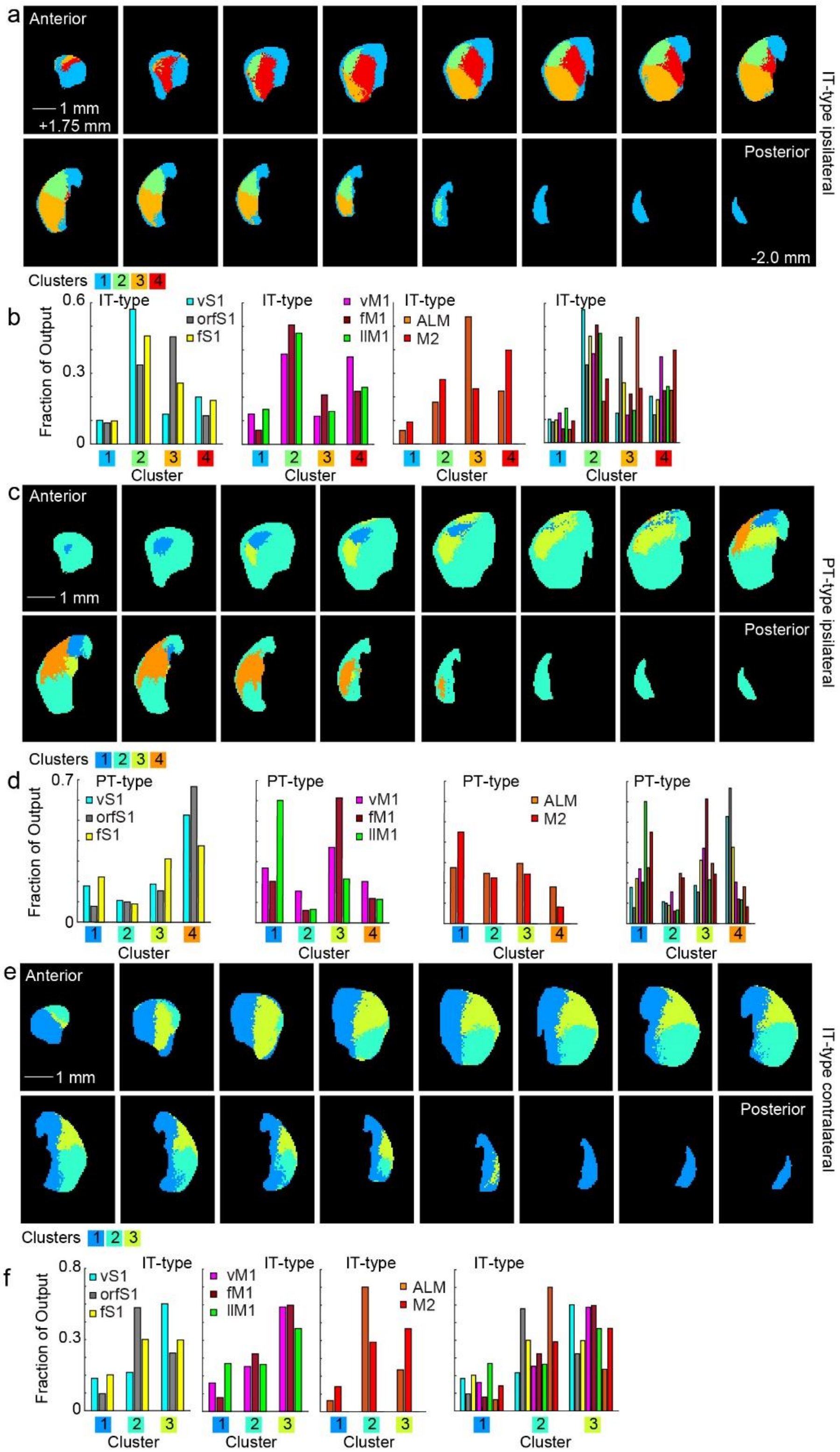
a
Anterior
1 mm
+1.75 mm
Posterior
-2.0 mm
IT-type ipsilateral
Clusters 1 2 3 4
b
IT-type
vS1
orfS1
fS1
vM1
fM1
llM1
ALM
M2
Fraction of Output
Cluster
c
Anterior
1 mm
Posterior
PT-type ipsilateral
Clusters 1 2 3 4
d
PT-type
vS1
orfS1
fS1
vM1
fM1
llM1
ALM
M2
Fraction of Output
Cluster
e
Anterior
1 mm
Posterior
IT-type contralateral
Clusters 1 2 3
f
IT-type
vS1
orfS1
fS1
vM1
fM1
llM1
ALM
M2
Fraction of Output
Cluster

Supplementary Figure 8 | Subdivision of ipsilateral striatum based on a subset of sensorimotor cortical projections

(a) *k*-means clustering of striatal pixels based on mean fluorescence intensity from each of the eight injection site clusters for only IT-type pyramidal neurons. The striatal clusters are illustrated as four colors (legend, at bottom) in evenly spaced planes every 0.25 mm from anterior (top left) to posterior (bottom right). Scale bar, 1 mm. (b) The fraction of the output from each of the eight injection site clusters to a given striatal division from IT-type projections. Graphs are divided into sensory (left), motor (center), and frontal (right), with all areas together at far right. (c-d) *k*-means clustering of striatal pixels based on mean fluorescence intensity from each of the eight injection site clusters for only PT-type pyramidal neurons. The striatal clusters are illustrated as four colors. (e-f) *k*-means clustering of contralateral striatal pixels based on mean fluorescence intensity from each of the eight injection site clusters for only IT-type pyramidal neurons. The striatal clusters are illustrated as three colors.
